# A laser capture microdissection‐based transcriptome analysis on salt glands highlights salt secretion mechanism in mangrove plant *Avicennia marina*

**DOI:** 10.1111/tpj.71143

**Published:** 2026-10-07

**Authors:** Ming‐Yue Wei, Ling Sun, Li‐Han Zhuang, Xing‐Yue Hong, Ze‐Jun Guo, Huan Li, Hezi Huang, Lu‐Dan Zhang, Dong‐Na Ma, Zhi‐Jun Shen, Xiu‐Xiu Wang, Xue‐Yi Zhu, Han‐Chen Tang, Hai‐Lei Zheng

**Affiliations:** ^1^ College of Ecology, Resources and Environment DeZhou University DeZhou Shandong 253000 P. R. China; ^2^ Key Laboratory for Subtropical Wetland Ecosystem Research of MOE, College of the Environment and Ecology Xiamen University Xiamen Fujian 361102 P. R. China; ^3^ Guangxi Laboratory on the Study of Coral Reefs in the South China Sea, Coral Reef Research Center of China, School of Marine Sciences Guangxi University Nanning 530004 P. R. China; ^4^ College of Food and Bio‐engineering Bengbu University Bengbu Anhui 233030 P. R. China; ^5^ Houji Laboratory in Shanxi Province Shanxi Agricultural University Taiyuan Shanxi 030000 P. R. China; ^6^ College of Food and Bioengineering Xuzhou University of Technology Xuzhou Jiangsu 221000 P. R. China; ^7^ Fujian Institute of Subtropical Botany Xiamen Fujian 361105 P. R. China

**Keywords:** mangrove, salt glands, laser capture microdissection, transcriptome, aquaporins, salt tolerance

## Abstract

Salt glands, developed in the epidermis of salt‐secreting mangrove plant *Avicennia marina* leaves and specialized in salt secretion, are critical for improving the survival under high saline habitats. However, the salt secretion mechanism in *A. marina* remains poorly understood. In this study, laser capture microdissection was employed to isolate salt glands from the upper epidermis of *A. marina*, followed by transcriptome sequencing (RNA‐Seq) to comparatively analyze gene expression profiles of salt glands under 0 mM NaCl and 400 mM NaCl treatments. Additionally, non‐invasive micro‐test technology (NMT) was used to measure real‐time Na^+^ and Cl^−^ efflux rates from individual salt glands; transmission electron microscopy (TEM) was applied to observe subcellular structural changes; and heterologous expression assays were performed for functional validation of candidate genes. Our results showed that 400 mM NaCl treatment led to an increase in Na^+^ and Cl^−^ fluxes from salt glands, with the Na^+^ efflux reaching 2389 pmol cm^−2^ s^−1^ and the Cl^−^ efflux reaching 1652 pmol cm^−2^ s^−1^. In addition, a significant increase in mitochondrial density and vesicle number in secretory cells was found in 400 mM NaCl treatment salt glands of *A. marina* leaves. LCM‐RNAseq identified a total of 1741 differentially expressed genes, which were significantly enriched in pathways related to vesicle trafficking, ion transport, compatible solute transport, aquaporins‐based water transport, hormone signaling, cell wall and cuticle wax biosynthesis. Functional validation demonstrated that *AmNHX2* (Na^+^/H^+^ antiporter), *AmCLC‐a* (chloride channel), and *AmSCAMP1* (secretory carrier membrane protein) significantly enhanced salt tolerance when heterologously expressed in yeast, tobacco, or Arabidopsis, and were involved in Na^+^/Cl^−^ transport or vesicle formation and trafficking. Collectively, this study reveals the molecular mechanisms by which salt glands of *A. marina* coordinately respond to salt stress through multiple pathways, including ion transport, vesicle trafficking, osmotic regulation, and cell wall plasticity, providing crucial insights for understanding salt tolerance in salt‐secreting mangrove plants.

## Significance Statement

Salt glands are critical for mangrove survival in saline habitats, yet their molecular secretion mechanisms remain poorly understood. By integrating laser capture microdissection‐based transcriptomics with ion flux measurements, ultrastructural analysis, and functional validation, this study reveals that ion transport, vesicle trafficking, osmotic regulation, and cell wall plasticity participate in regulating salt secretion, providing crucial insights into salt tolerance in salt‐secreting mangrove plants.

## INTRODUCTION

Coastal saline ecosystems, including salt marshes and mangrove forests, host plants that must cope with high salinity (Liu et al., 2023; Zhao et al., 2024) and salt‐secreting mangrove plants have adapted to grow in high saline environments. They have evolved various adaptive strategies such as salt exclusion at the roots and salt secretion in the leaves (Balsamo et al., 1995; Parida & Jha, 2010). Based on the presence or absence of salt glands, mangrove plants are broadly classified into the types of salt‐excluding and salt‐secreting plants. Salt‐excluding mangroves eliminate excess salt through ultrafiltration in the root cortex cells (Wang et al., 2002). For example, the salt concentration of the xylem sap of the salt‐excluding mangrove such as *Rhizophora mangle* is only one‐tenth of that of seawater through ultrafiltration by the root cell membrane (Lawton et al., 1981). In addition to salt exclusion by the root system, salt‐secreting mangrove plants can also improve their salt tolerance by using salt glands, a special salt‐secreting structure in the leaves (Berlyn, 1986). For example, the salt‐secreting ability of three salt‐secreting mangrove plants is *A. marina* > *Aegiceras corniculatum* > *Acanthus ilicifolius*, which is consistent with the order of their salt tolerance, suggesting the important role of leaf salt glands in mediating salt tolerance for salt‐secreting mangrove plants (Ye et al., 2005).

It has been shown that salt secretion from the salt glands is an energy‐consuming process and the H^+^‐ATPase and Na^+^/H^+^ antiporter participate in the Na^+^ secretion from the salt glands (Chen et al., 2010; Dschida et al., 1992). For example, the H^+^‐ATPase in *A. germinans* salt glands is distributed in the cytoplasmic membrane of secretory cells, while inhibitors of H^+^‐ATPase can eventually restrain the Na^+^ secretion from the salt glands (Dschida et al., 1992). Guo et al. (2020) demonstrated that the plasma membrane Na^+^/H^+^ antiporter (SOS1) is involved in Na^+^ secretion from the salt glands of *Karelinia caspia*, and silencing of *KcSOS1* resulted in a reduced rate of Na^+^ secretion from the salt glands. In addition, after salt treatment, the transcription level of vacuole membrane Na^+^/H^+^ antiporter (NHX) in salt bladders of *Chenopodium quinoa* was significantly up‐regulated, and thereafter the salt removal was also increased (Bohm et al., 2018). Recently, Ramakrishna et al. (2025) reported that SOS1 strongly accumulated at endomembrane vesicular systems by the use of new genomic, complementing fluorescently tagged SOS1 variants and CryoNanoSIMS, supporting a role of SOS1 in vacuolar Na^+^ sequestration.

Water conservation and compatible solutes accumulation are also important during the salt secretion via salt glands (Adolf et al., 2013; Agarie et al., 2007; Feng et al., 2015; Tan et al., 2013). Adolf et al. (2013) suggested salt bladders may play an important role as a secondary epidermis to reduce water loss in *Chenopodium quinoa* leaves. Under salt stress, pinitol accumulates to over 9% of dry matter, of which the concentration exceeds 700 mM in *Mesembryanthemum crystallinum* leaves (Agarie et al., 2007). In addition, K^+^ accumulations in the cytoplasm and nucleus were also reported in the *Limonium bicolor* of salt gland cells (Feng et al., 2015).

*A. marina* is one of the most salt‐tolerant mangrove plants, and has adapted to grow and reproduce in habitats with seawater salinity ranging from 10 to 35‰, and its leaf surfaces are often covered with salt crystals (Nguyen et al., 2015). In *A. marina*, salt glands consist of collecting cells, stalk cells, secretory cells, and cuticle (Dassanayake & Larkin, 2017). Salt exclusion by gland secretion is efficient, thus holding a relative lower concentration of salt ions in the sensitive parts above‐ground shoots (Guo et al., 2023). In the previous study, we revealed that nitric oxide (NO) enhances salt secretion of *A. marina* by enhancing the accumulations of H^+^‐ATPase and Na^+^/H^+^ antiporter proteins in whole leaf tissues (Chen et al., 2010). Recently, Ding et al. (2024) found the Na^+^ and Cl^−^ content in salt gland exudates were enhanced and the Na^+^ fluxes from salt glands were suppressed by Na^+^/H^+^ antiporter inhibitor in salt‐treated *A. marina* plants. Besides, Guo et al. (2023) reported Na^+^/H^+^ antiporter genes, H^+^‐ATPase genes, and K^+^ channel genes were up‐regulated in the adaxial epidermis of the *A. marina* leaves under salt treatment. By using the epidermis of another mangrove, *A. officinalis*, Jyothi‐Prakash et al. (2014) found that *AoDHN1* gene encoding dehydrin 1 protein plays an important role in salt tolerance. Since the materials used in the above‐mentioned studies were mainly from the leaves or epidermis rather than the salt glands themselves, it is difficult to accurately evaluate the role of salt glands in salt tolerance. Particularly, the gene expression profiling in the salt glands induced by salt is not clear yet. Recently, laser capture microdissection (LCM) coupled with RNA‐Seq technology enables sensitive and genome‐wide analysis of gene expression in specific tissues or cells (Jardinaud et al., 2016). However, it has not been reported using LCM‐RNAseq to analyze the salt secretion mechanism in the salt glands.

In this study, a combined scanning electron microscopy (SEM) coupled with X‐ray emission spectrum, inductively coupled plasma‐mass spectrometry (ICP‐MS), and non‐invasive micro‐test technology (NMT) were employed to investigate the salt secretion mechanism in the salt glands of *A. marina* leaves exposed to NaCl, including salt secretion phenotype, Na^+^ and Cl^−^ content in the leaves, Na^+^ and Cl^−^ secretion rate, as well as Na^+^ and Cl^−^ flux from the salt glands. Further, transmission electron microscopy (TEM) was used to compare the cytological differences in the salt glands between the control and the salt‐treated plants. In addition, LCM‐RNAseq was used to analyze the transcriptomic signatures of the salt glands under salt treatment. Lastly, the functional validations for three selected differentially expressed genes including *AmNHX2*, *AmCLC‐a*, and *AmSCAMP1* using heterogeneous expression systems such as yeast, tobacco, and Arabidopsis suggests that these genes are closely related to the salt ion transport and vesicle trafficking associated salt tolerance. This study may provide a perspective on how salt glands function in mangrove *A. marina*.

## RESULTS

### Effects of NaCl on leaf salt secretions of *A. marina*

To study the effect of NaCl on salt secretion in *A. marina* leaves, salt crystals deposited on the leaf surface of *A. marina* under different concentrations of NaCl (0–600 mM) were observed (Figure 1A). More salt crystals were observed on the leaf surface as NaCl concentration increased. The salt crystals were most apparent when the seedlings were treated with 400 mM NaCl for 30 days (Figure 1A). However, the salt crystals on the leaf surface decreased under 600 mM NaCl treatment. SEM‐EDAX analysis confirmed the salt crystals are mainly comprised of sodium and chloride (Figure 1B,C). Further results showed that Na^+^ and Cl^−^ secretion rates reached the maximum under 400 mM NaCl treatment, which were 57.5 mg m^−2^ day^−1^ (Figure 1D) and 39.9 mg m^−2^ day^−1^ (Figure 1F), respectively. In addition, NaCl treatment resulted in a significant increase of Na^+^ and Cl^−^ content in the leaves of *A. marina* compared to the control (CK) plants (Figure 1E,G).

**Figure 1:**
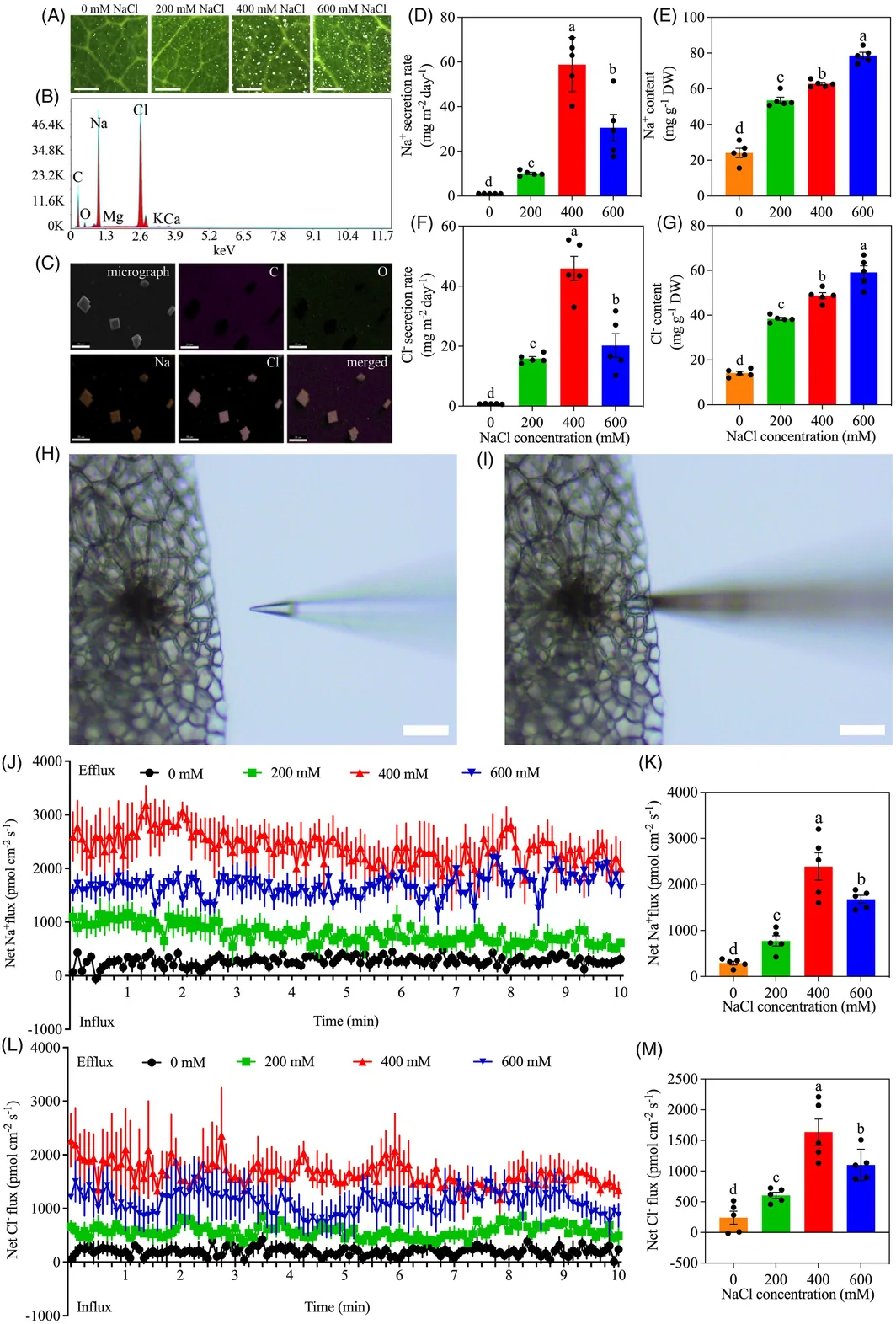
Physiological response of *A. marina* seedlings treated with different concentrations of NaCl for 30 days. (A) Comparison of salt crystals on adaxial surfaces treated with 0, 200, 400 and 600 mM NaCl for 30 days. Scale bars = 1 mm. (B) Scanning electron microscopy (SEM) coupled with X‐ray emission spectrum analysis of salt crystals from 400 mM NaCl‐treated plants. Na and Cl are the dominant elements of salt crystals. (C) SEM micrographs and elements (carbon, C; oxygen, O; sodium, Na; and chloride, Cl) maps of salt crystals from 400 mM NaCl‐treated plants. Scale bars = 20 μm. (D) The Na^+^ secretion rate in the adaxial leaf surface. (E) Na^+^ content in the leaves. (F) The Cl^−^ secretion rate in the adaxial leaf surface. (G) Cl^−^ content in the leaves. (H) The non‐invasive ion‐selective electrode was close to the adaxial surface of the leaf. (I) The non‐invasive ion‐selective electrode was moved on the salt gland and the focal plane focused on the leaf surface. (J) Net Na^+^ flux record in salt glands of *A. marina* leaves treated with 0, 200, 400 and 600 mM NaCl for 30 days. (K) Calculated average net Na^+^ fluxes from (I). (L) Net Cl^−^ flux record in salt glands of *A. marina* leaves treated with 0, 200, 400 and 600 mM NaCl for 30 days. (M) Calculated average net Cl^−^ flux from (I). Scale bar of h and i = 50 μm. Values of (D), (E), (F), (G), (K) and (M) represent the mean ± SE of five biological replicates. Significant differences (*P* < 0.05) are indicated by different letters, according to one‐way ANOVA.

### Effects of NaCl on Na^+^ and Cl^−^ fluxes from the salt glands

To understand how the salt gland responds to different salinities, Na^+^ and Cl^−^ fluxes from the salt glands were measured using the NMT (Figure 1H,I). As shown in Figure 1J–M, the Na^+^ and Cl^−^ efflux from the salt glands of the CK plants was maintained at a very low level, approximately 273 pmol cm^−2^ s^−1^ and 198 pmol cm^−2^ s^−1^, respectively. Under the treatment of 400 mM NaCl, the Na^+^ and Cl^−^ efflux rates of the salt glands reached the maximum, which were 2389 pmol cm^−2^ s^−1^ and 1652 pmol cm^−2^ s^−1^, respectively (Figure 1J–M). Taken together, the salt secretion rates and the Na^+^ and Cl^−^ effluxes were significantly high under 400 mM NaCl treatment. Therefore, 400 mM NaCl was selected for the subsequent experiments.

### Transmission electron microscopy analysis of the salt glands

As shown in Figure 2, the salt glands are located in the epidermis of *A. marina* leaves. Figure 2A–C shows salt glands of *A. marina* leaves under confocal microscopy. Figure 2A shows the general morphology of salt glands on the leaf surface in a bright field. Figure 2D,E shows salt glands of *A. marina* leaves under scanning electron microscopy. The salt glands are surrounded by substances with autofluorescence (Figure 2B,C). The salt crystals secreted by the salt glands are deposited on the leaf surface with approximately 30–40 μm in diameter (Figure 2D,E). Figure 2F,G show the salt glands consist of collecting cells, stalk cells, and secretory cells.

**Figure 2:**
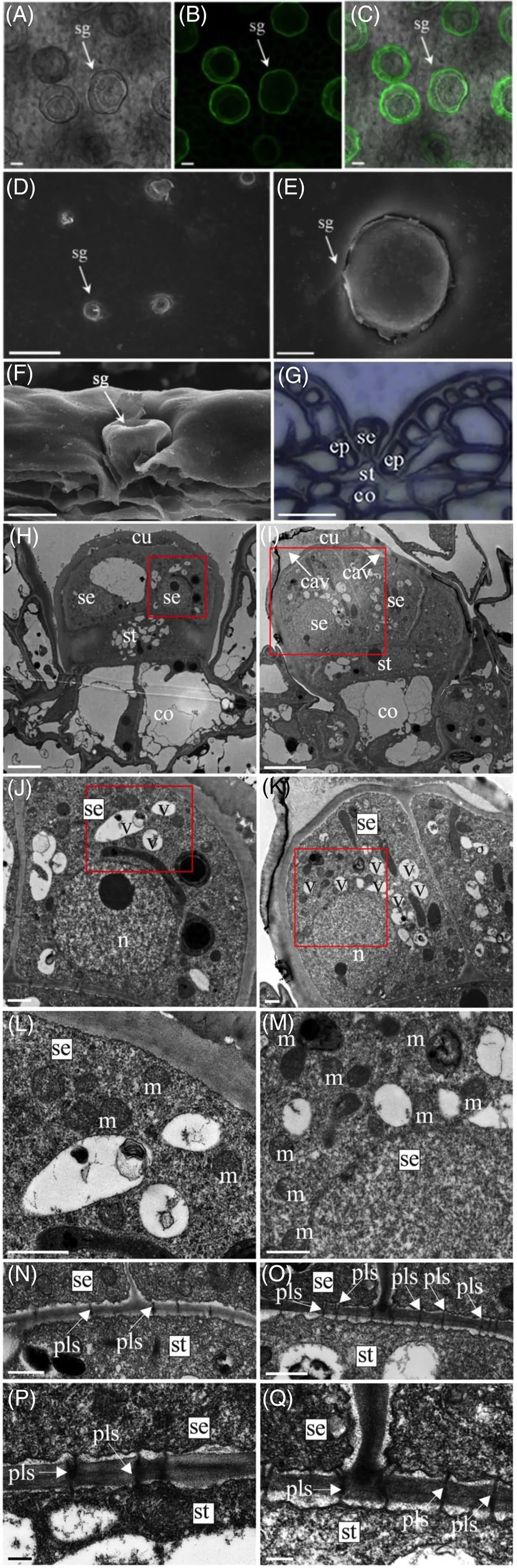
Salt gland structure and ultrastructure under control and stress conditions. (A–C) Morphology of the salt glands under a dissecting microscope. The bright field (A), dark field (B), and merge of the bright field and dark field (C). The excitation wavelength was 488 nm (8–10%) and optical sections were taken using Leica SP8. Images from optical sectioning were stacked to give a clearer picture of the salt glands. (D,E) Morphology of the salt glands under scanning electron microscopy. (F) Side view of salt gland. (G) The structure of salt gland. (H,G,L,N,P) Transmission micrographs of the salt gland under control. (I,K,M,O,Q) Transmission micrographs of the salt gland from 400 mM NaCl‐treated *A. marina* plant leaves. J is the enlargement of the red box in h. k is the enlargement of the red box in (I). (L) is the enlargement of the red box in (J). (M) is the enlargement of the red box in (K). cav, subcuticular collecting cavity; co, collecting cell; cu, cuticle layer; ep, epidermal cell; m, mitochondria; n, nucleus; pls, plasmodesmata; se, secretory cell; sg, salt gland; st, stalk cell; v, vesicle. Scale bars: A, B, C, E = 10 μm; D = 100 μm; F = 20 μm; G = 40 μm; H, I = 5 μm; J, K, L, M, N, O = 1 μm; P, Q = 250 nm.

The transmission electron microscope observations from the CK and salt‐treated plants revealed that the salt glands of *A. marina* leaves are multicellular (Figure 2H–Q). The multicellular salt gland is composed of secretory cells, stalk cell, collecting cells and cuticle layer (Figure 2H,I). The salt glands of NaCl‐treated plants showed some ultrastructural changes compared with the CK. The upper portion of the glands is entirely enclosed by a cuticle layer, and the subcuticular collecting cavity is located between the secretory cells and the cuticle layer in NaCl‐treated plants compared with the CK plants (Figure 2H,I). There are more vesicles in the secretory cells of the salt glands in NaCl‐treated plants compared with the CK plants (Figure 2J,K). Secretory cells from NaCl‐treated plants exhibited an increase in mitochondria density (Figure 2L,M). In addition, the plasmodesmata between the stalk cell and the secretory cells are more abundant in NaCl‐treated plants compared with the CK plants (Figure 2N–Q).

### LCM‐RNAseq analysis of salt glands in response to NaCl treatment

To reveal possible candidate genes regulating salt secretion in the salt glands, LCM (Figure S1) coupled with RNA‐Seq (Table S1) was used to analyze the transcriptomes of the isolated salt gland samples. The specimens were observed under a microscope after removing the lower epidermis and mesophyll. The intact salt glands with almost no contamination by other cells were obtained (Figure S1, Figure 2). Figure S1 showed the application of LCM for isolation of salt glands in *A. marina* leaf sections. Young leaves were fixed, embedded in optimum cutting temperature compound, and 20 μm thick sections were produced. Salt gland cells were easily distinguishable from other cells by their morphological features. For example, pictures from a to d in Figure S1 showed the harvest process of salt gland cells from gland‐rich epidermis. A total of 4.4–7.1 million raw reads for each sample were generated (Table S1). After removing low‐quality sequences, adaptor sequences and sequence contaminants, 4.2–6.6 million cleaned reads were mapped to the *A. marina* genome (Table S1). The Q20 and Q30 values are 91.74–93.98% and 91.28–92.30%, respectively, and the GC content was 40.28–42.29% (Table S1).

### GO enrichment analysis of differentially expressed genes

A set of 1741 differentially expressed genes (DEGs) was identified based on adjusted *P* < 0.05 and log_2_ fold change >1, with 1053 up‐regulated and 688 down‐regulated genes (Figure S2; Table S2). An enrichment analysis of Gene Ontology (GO) functional categories among the DEGs was performed, revealing a predominance of transport (GO: 0006810), response to stimulus (GO: 005086), carbohydrate metabolic process (GO: 0005975), and lipid metabolic process (GO: 0006629) (Figure S3).

BinGO was used to visualize networks of GO terms enriched among DEGs (Figure 3). Transport GO term consists of genes annotated as ion transport, transmembrane transport, carbohydrate transport, water transport, organic acid transport, amine transport, and vesicle‐mediated transport, especially sodium ion transport, water transport, glucose transport, and vesicle trafficking (Figure 3A). Response to stimulus GO term consists of genes annotated as response to abiotic stress and response to chemical stress, especially hormone stimulus, water deprivation, and osmotic stress (Figure 3B). Carbohydrate metabolic GO term consisted of genes related to cell wall metabolic process such as cellulose metabolic process, beta‐glucan metabolic process, and polysaccharide biosynthesis process (Figure 3C). Lipid metabolic GO term consisted of genes related to cuticle wax biosynthesis process such as cuticle biosynthesis process, suberin biosynthetic process, and very long‐chain fatty acid biosynthetic process (Figure 3D). Based on GO enrichment analysis, we focused on the gene families related to ion transport, vesicle trafficking, water and compatible solutes transport, hormone response, cell wall, and cuticle wax biosynthesis process.

**Figure 3:**
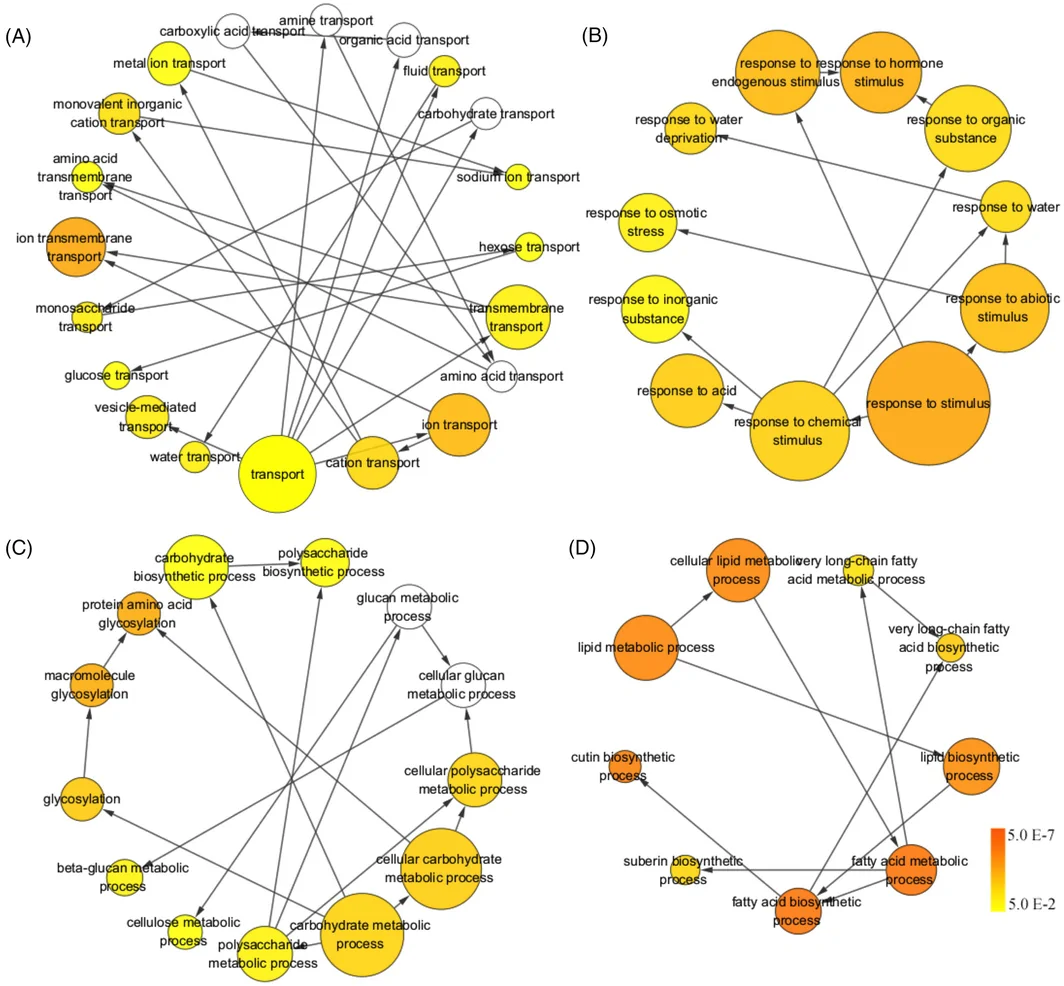
Networks of enriched gene ontology (GO) terms among differentially expressed genes (DEGs) by NaCl treatment in the *A. marina* salt gland transcriptome. (A) Transport GO term, (B) Response to stimulus GO term, (C) Carbohydrate metabolic process GO term, and (D) Lipid metabolic process GO term. Enriched GO terms were identified using BINGO and visualized with Cytoscape, as described in the Materials and Methods. GO terms are connected based on their ancestor–child relationships. The colors of circles indicate the *P* value of enrichment. Sizes of circles represent the size of GO terms in the background TAIR10 annotation.

### DEGs involved in ion transport and vesicle trafficking

Enrichment analysis showed that 23 DEGs were involved in ion transport (Figure 4A). These genes all showed up‐regulation in NaCl‐treated seedling samples. Of these, eighteen genes were involved in Na^+^ transport, including Na/H^+^ antiporter (NHX/SOS1), potassium transporter (AKT), H^+^‐ATPase or its subunits, pyrophosphate‐energized vacuolar membrane proton pump (PEPP), and cyclic nucleotide‐gated ion channel (CNGC). Five genes were involved in Cl^−^ transport, including chloride channel (CLC) and nitrate transporter (NRT1).

**Figure 4:**
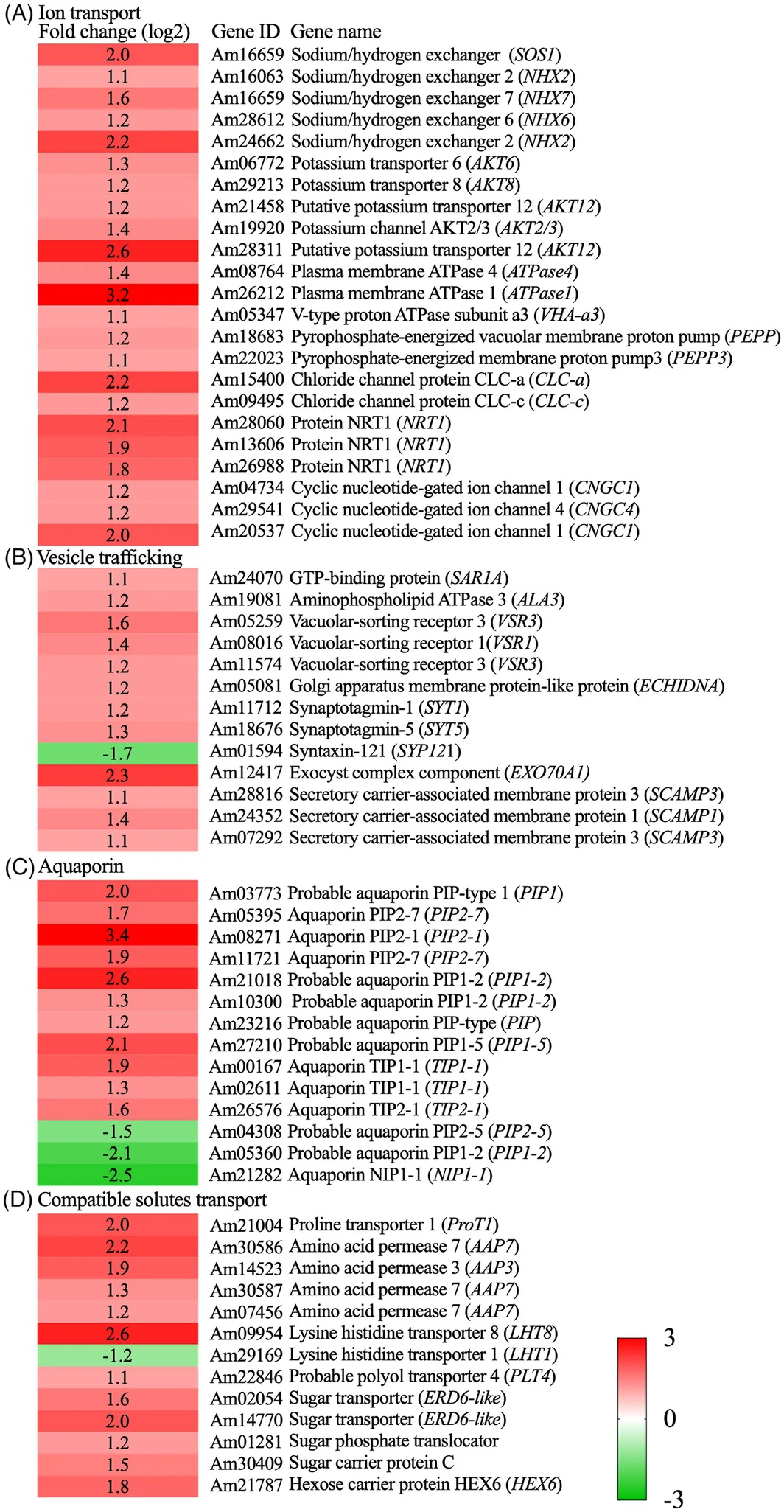
Selected genes related to (A) ion transport, (B) vesicle trafficking, (C) water and (D) compatible solutes transport. The up‐ or down‐regulated DEGs are represented in red or green. The color bar represents the intensity of gene expression. The color scale ranges from −3 to 3, representing log_2_ fold changes in gene expression. Values beyond this range (>3 or <−3) are shown in the darkest red or green, respectively.

Thirteen DEGs were involved in vesicle trafficking, while most of them were up‐regulated in NaCl‐treated seedling samples (Figure 4B). Among those, GTP‐binding protein (SAR1A) and aminophospholipid ATPase 3 (ALA3) were involved in vesicle budding. Four genes were involved in vesicle transport, including three genes encoding vacuolar‐sorting receptor (VSR), and one gene encoding Golgi apparatus membrane protein‐like protein (ECHIDNA). Seven genes were involved in the fusion process between vesicles and cell membrane, including one gene encoding syntaxin‐121 (SYP121), two genes encoding synaptotagmin (SYTs), one gene encoding exocyst complex component (EXO70A1), and three genes encoding secretory carrier‐associated membrane protein (SCAMPs).

### DEGs involved in water and compatible solutes transport

Fourteen DEGs encode aquaporin and most of them were up‐regulated in NaCl‐treated seedling samples (Figure 4C). Among them, eleven genes encode plasma membrane intrinsic proteins (PIPs) and three genes encode tonoplast intrinsic proteins (TIPs/NIP1‐1).

Thirteen DEGs were involved in compatible solutes transport, and most of them were up‐regulated in NaCl‐treated seedling samples (Figure 4D), among which 7 genes were involved in the transport of proline, including one proline transporter (ProT1), four amino acid permease (AAPs), and two lysine‐histidine transporters (LHTs). The other six genes are involved in polyol and sugar transport, including polyol transporter 4 (PLT4), sugar transporter, and hexose carrier protein (HEX6).

### DEGs involved in hormone signaling

Seventeen DEGs were involved in hormone signal transduction (Figure 5A). These genes were mainly involved in abscisic acid (ABA) and ethylene signaling. Six genes were involved in ABA signal transduction. Among them, 9‐cis‐epoxycarotenoid dioxygenase (NCED1) genes involved in ABA biosynthesis were down‐regulated, while ABA receptor (PYL4) genes were also down‐regulated. On the contrary, ABA 8‐hydroxylase 1 (CYP707A) genes encoding key enzymes in ABA degradation were up‐regulated.

**Figure 5:**
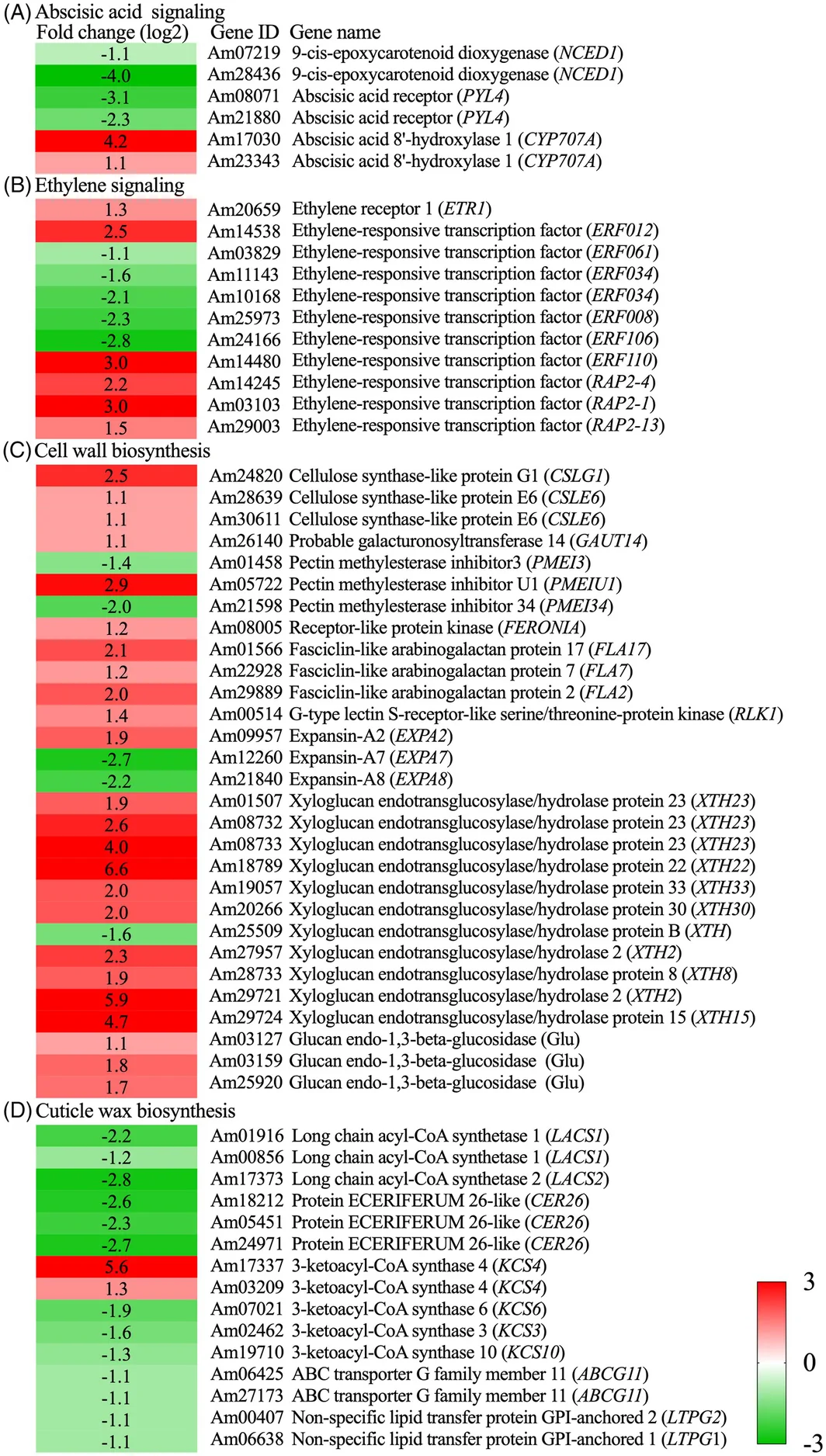
Selected genes related to (A) abscisic acid signaling, (B) ethylene signaling, (C) cell wall, and (D) cuticle wax biosynthesis. The up‐ or down‐regulated DEGs are represented in red or green. The color bar represents the intensity of gene expression. The color scale ranges from −3 to 3, representing log_2_ fold changes in gene expression. Values beyond this range (>3 or <−3) are shown in the darkest red or green, respectively.

Eleven genes were involved in ethylene signal transduction (Figure 5B). Among them, one gene encoding ethylene receptor 1 (ETR1) was up‐regulated. Six out of seven ERFs genes encoding ethylene responsive transcription factor were down‐regulated. However, all four RAPs genes encoding another type of ethylene responsive transcription factor were up‐regulated in NaCl‐treated seedling samples.

### DEGs involved in the biosynthesis processes of cell wall and cuticle wax

Twenty‐nine DEGs were identified that involve genes in cell wall biosynthesis (Figure 5C). Of these, there are genes related to cellulose and pectin biosynthesis, such as genes encoding cellulose synthase‐like protein (CSLs), fasciclin like arabinogalactan protein 17 (FLA17), and pectinesterase inhibitor (PMEIs). The other genes were involved in cell wall ductility, such as genes encoding xyloglucan hydrolase protein (XTHs), glucan endo‐1,3‐beta‐glucosidase (Glu), and expansin (EXPs). Most of these genes were up‐regulated in NaCl‐treated plants.

Fifteen genes were involved in cuticle wax biosynthesis (Figure 5D). Most of these genes showed down‐regulation in NaCl‐treated plants, such as genes encoding long‐chain acyl‐CoA synthetase (LACS), protein ECERIFERUM 26‐like (CER26), 3‐ketoacyl‐CoA synthase (KCS), ABC transporter G family member 11 (ABCG11), and non‐specific lipid transfer protein GPI‐anchored (LTPG).

### Functional validations of selected DEGs related to ions transport and vesicles trafficking in yeast

In order to verify the roles of selected DEGs identified from the salt‐induced transcriptome of *A. marina* salt glands in salt tolerance, three DEGs including *AmNHX2*, *AmCLC‐a* and *AmSCAMP1* were applied to perform the functional validation experiments. Firstly, using transgenic yeast cells (BY4741 line), the empty vector and transgenic yeasts grew as a similar pattern on the glucose plate (Figure 6A), while all three transgenic yeasts grew well on the galactose plate compared with the empty vector under 500 mM NaCl treatment (Figure 6B), indicating those three selected DEGs play important roles in salt tolerance. Secondly, the salt ions accumulation assay using the above transgenic yeast cells transformed by *AmNHX2* and *AmCLC‐a* (Figure 6C,D) revealed that the contents of Na^+^ and Cl^−^ in the transgenic yeast cells under salt treatments can be altered by the two selected ions transport genes, implying those genes do play salt ions transport functions. Thirdly, using Na^+^ transport deficient yeasts (AXT3K line), the role of *AmNHX2* in Na^+^ transport was further analyzed. *AmNHX2* overexpressing yeasts (AXT3K line which is the mutant in Na^+^ transport) on the galactose plate plus with 15 mM NaCl showed well growth than that of empty yeasts (Figure 6E). Additionally, the content of Na^+^ in AXT3K yeasts cells transformed by *AmNHX2* is correspondingly higher than that in the empty yeast under 15 mM NaCl treatment. Above results imply that the selected three DEGs identified from the salt‐induced transcriptome of *A. marina* salt glands play roles in salt tolerance.

**Figure 6:**
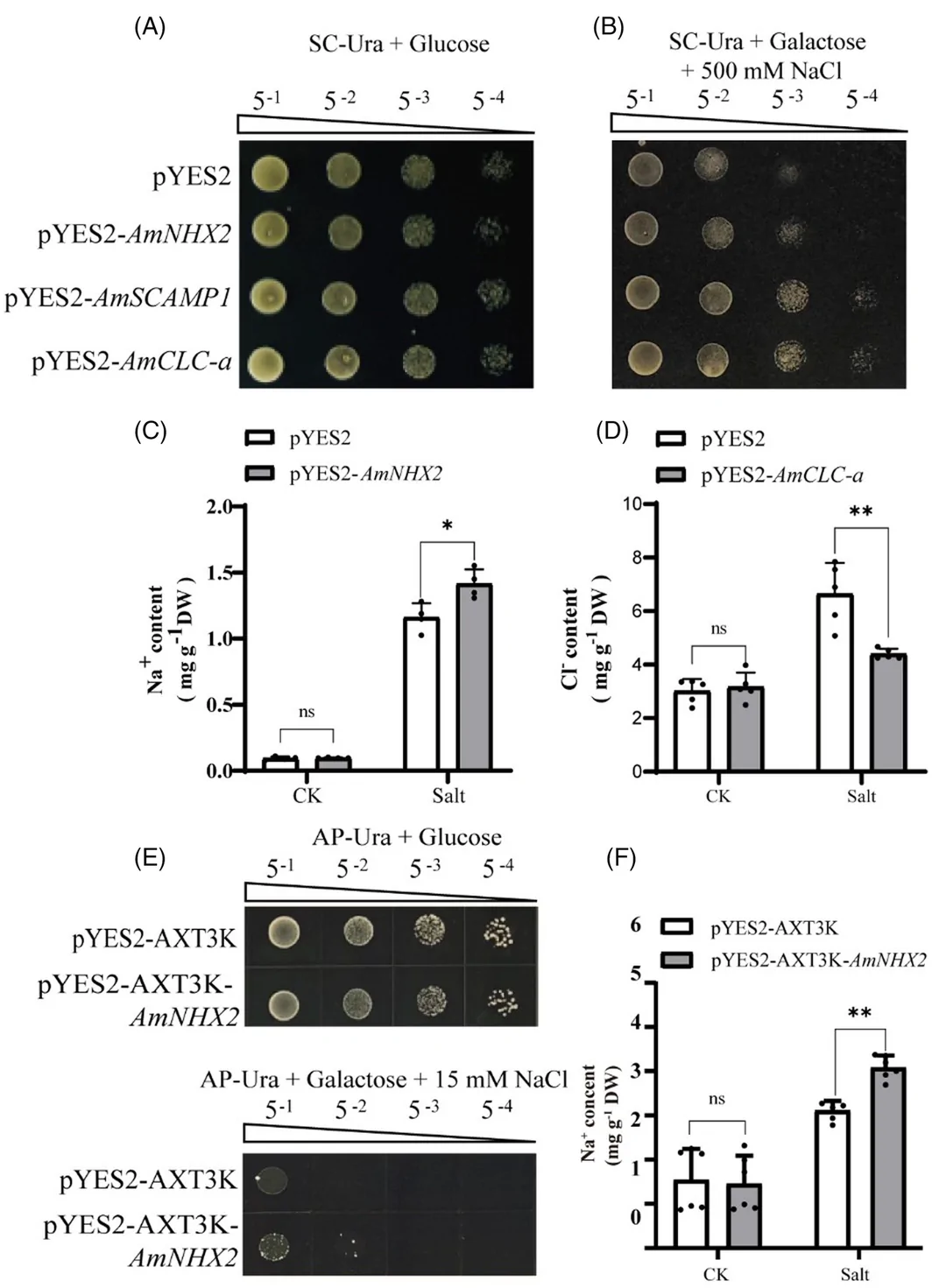
Salt tolerance assay using transgenic yeast cells. First, three differentially expressed genes including *AmNHX2, AmCLC‐a*, and *AmSCAMP1* identified from salt glands transcriptome were selected to verify their roles in salt tolerance using BY4741 yeasts. (A) The growth patterns of empty and transgenic yeasts (BY4741 line) on the glucose plate. (B) The growth patterns of empty and transgenic yeasts (BY4741 line) on the galactose plate under 500 mM NaCl treatment. (C) The contents of Na^+^ in the empty and BY4741 yeast cells transformed by *AmNHX2* under 500 mM NaCl. (D) The contents of Cl^−^ in the empty and BY4741 yeast cells transformed by *AmCLC‐a* under 500 mM NaCl. Second, using Na^+^ transport deficient yeast (AXT3K line), the role of *AmNHX2* in Na^+^ transport was analyzed. (E) The growth patterns of empty and transgenic yeast (AXT3K line) on the glucose plate and on the galactose plate with 15 mM NaCl. (F) The contents of Na^+^ in the empty and AXT3K yeast cells transformed by *AmNHX2* under 15 mM NaCl. The data are the mean ± SE. Asterisks represent significant differences between empty vector and transgenic yeast cells (**P < 0.01, *P < 0.05).

### Subcellular localization and functional validation of selected ions transport genes using tobacco leaf

Subcellular localization analyses of AmNHX2 and AmCLC‐a using tobacco epidermis cells showed that AmNHX2 and AmCLC‐a are localized to the membrane system (Figure 4S). Next, we used tobacco leaf to validate the functions of the two selected genes *AmNHX2* and *AmCLC‐a* in salt ions transport. The salt ions accumulation assay using *AmNHX2*‐ and *AmCLC‐a*‐overexpressing tobacco leaves (Figure 7) revealed that the contents of Na^+^ and Cl^−^ in the transgenic tobacco leaves can be significantly regulated by the two selected ions transport genes under salt treatments, implying their important roles in salt ions transport functions.

**Figure 7:**
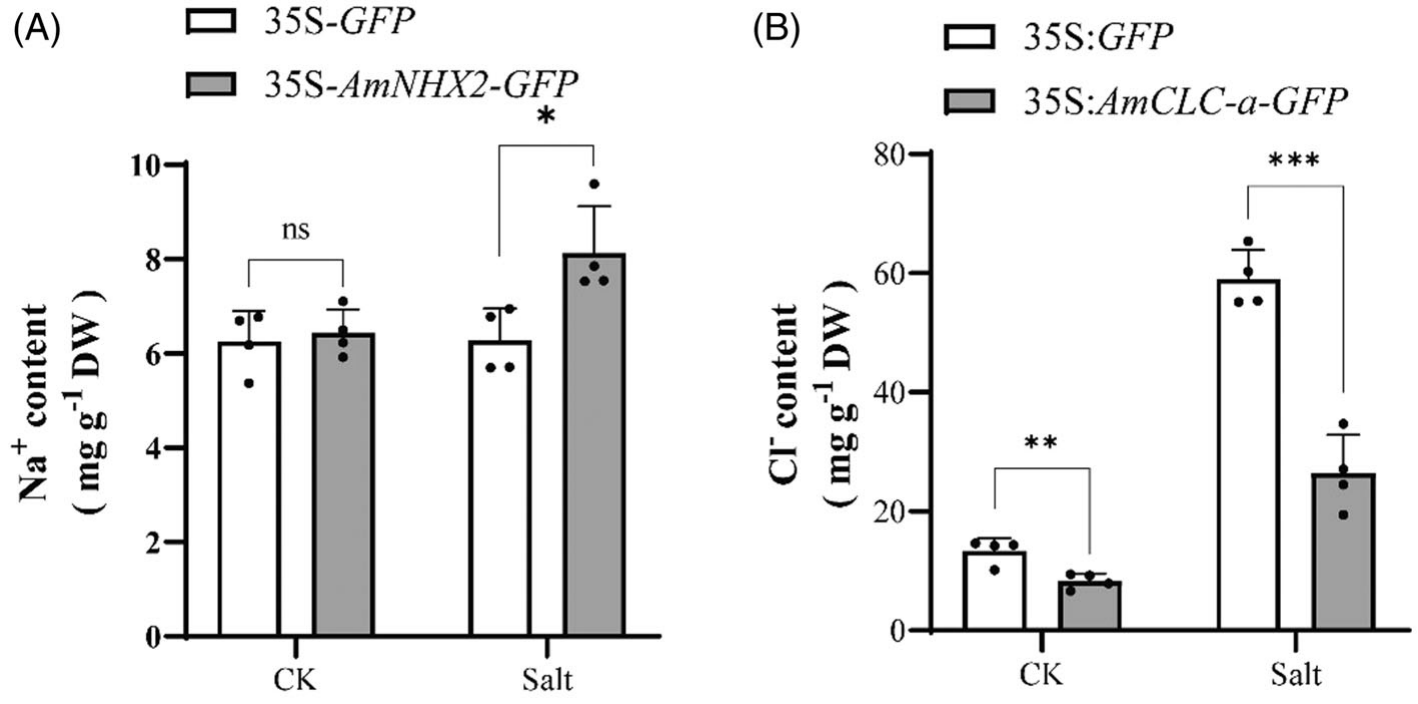
Ions accumulation assay using transgenic tobacco leaves. (A) The content of Na^+^ in the transgenic tobacco leaves transformed by *AmNHX2* under 300 mM NaCl. (B) The content of Cl^−^ in the transgenic tobacco leaves transformed by *AmCLC‐a* under 300 mM NaCl. The data are the mean ± SE. Asterisks represent significant differences between empty vector and transgenic tobacco leaves (***P < 0.001, **P < 0.01, *P < 0.05).

### Functional validations of *AmSCAMP1* in Arabidopsis

To explore the role of vesicle trafficking‐related gene *AmSCAMP1*, the functional validation analysis of *AmSCAMP1* was conducted using transgenic *A. thaliana*. After *AmSCAMP1* was transformed into *A. thaliana*, we performed the salt tolerance analyses. First, the root lengths of wild‐type (Col‐0) and *AmSCAMP1*‐overexpressed *A. thaliana* lines (OE2 and OE3) were measured, showing the significantly long root length in OE2 and OE3 than that in Col‐0 under 150 mM NaCl (Salt) treatment, while no root length difference between OE lines and Col‐0 was observed under the condition control (Figure 8A,B). Similar results were obtained in the fresh weight of Col‐0 and transgenic lines (OE2 and OE3) grown in soil with 250 mM NaCl treatment (Figure 8C). Besides, the measurement of Na^+^ fluxes from the root of transgenic lines (OE2 and OE3) using NMT revealed more efflux of Na^+^ in the root under salt treatment (Figure 8D,E). Correspondingly, the contents of Na^+^ in the whole plant of transgenic *A. thaliana* (OE2 and OE3) were much lower than that in Col‐0 under salt treatment (Figure 8F). In addition, the expression level of Na^+^ transport gene (*AtSOS1*) in *AmSCAMP*‐overexpressed *A. thaliana* lines (OE2 and OE3) exhibited profoundly higher than that in Col‐0 under NaCl treatment (Figure 8G).

**Figure 8:**
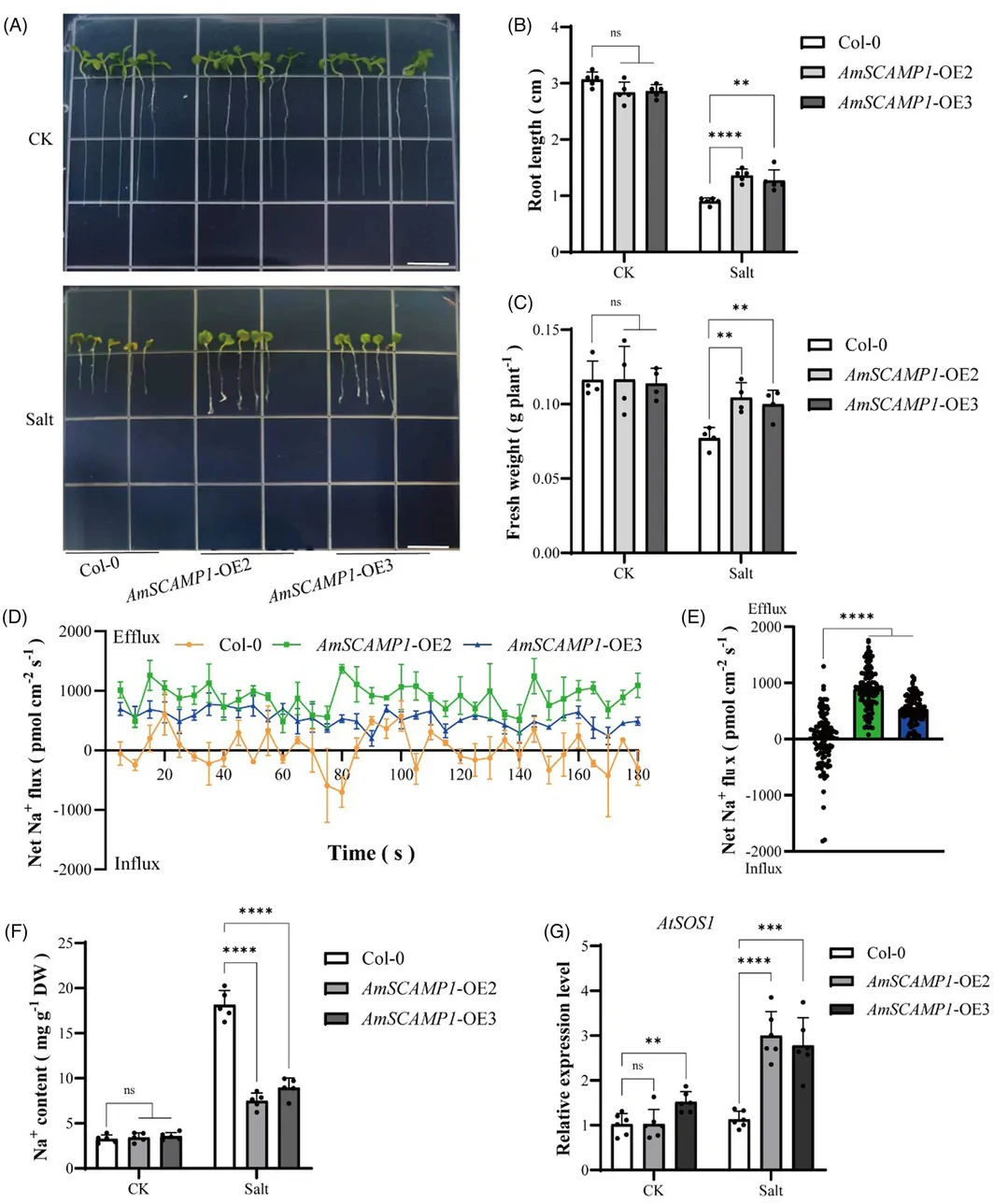
Functional analysis of *AmSCAMP1* in transgenic *A. thaliana* for salt tolerance. (A) Photographs of root lengths in wild‐type (Col‐0) and *AmSCAMP1*‐overexpressed *A. thaliana* lines (OE2 and OE3). Seeds of the indicated plants were germinated on 1/2 MS medium for 4 days, and then transferred to 1/2 MS medium without (CK) or with 150 mM NaCl (Salt) for another 4 days. Scale bars represent 1 cm. (B) Statistical analysis of root length of the plants shown in (A). (C) Fresh weight of Col‐0 and transgenic *A. thaliana* (OE2 and OE3) grown in soil with 250 mM NaCl. Seeds of the indicated plants were germinated on 1/2 MS medium for 7 days, and then the seedlings were transplanted to soil for another 7 days, then following treated without (CK) or with 250 mM NaCl (Salt) for 14 days. (D) The measurement of Na^+^ fluxes from the root of transgenic *A. thaliana* (OE2 and OE3) under 150 mM NaCl treatments using NMT. (E) Mean net Na^+^ fluxes calculated from (D). (F) The content of Na^+^ in the whole plant of Col‐0 and transgenic *A. thaliana* (OE2 and OE3) grown in soil with 250 mM NaCl. (G) The expression level of ion transport related genes (*AtSOS1*) in Col‐0 and *AmSCAMP*‐overexpressed *A. thaliana* lines (OE2 and OE3) under NaCl treatment. The data are the mean ± SE with at least three replicates. Asterisks represent significant differences between Col‐0 and AmSCAMP1‐overexpressed *A. thaliana* lines (OE2 and OE3) (****P < 0.0001, ***P < 0.001, **P < 0.01, *P < 0.05).

Then, the vesicles in the transgenic *A. thaliana* leaves treated with 150 mM NaCl were observed under TEM (Figure 9). Compared with those cellular ultrastructure of wild‐type (WT) and AmSCAMP1 OE under 0 mM (Figure 9A,B) and 150 mM (Figure 9C,D) NaCl treatments, AmSCAMP1 OE treated with 150 mM NaCl exhibits the most vesicles, suggesting *AmSCAMP1* contributes to cellular vesicle formation and trafficking under salt.

**Figure 9:**
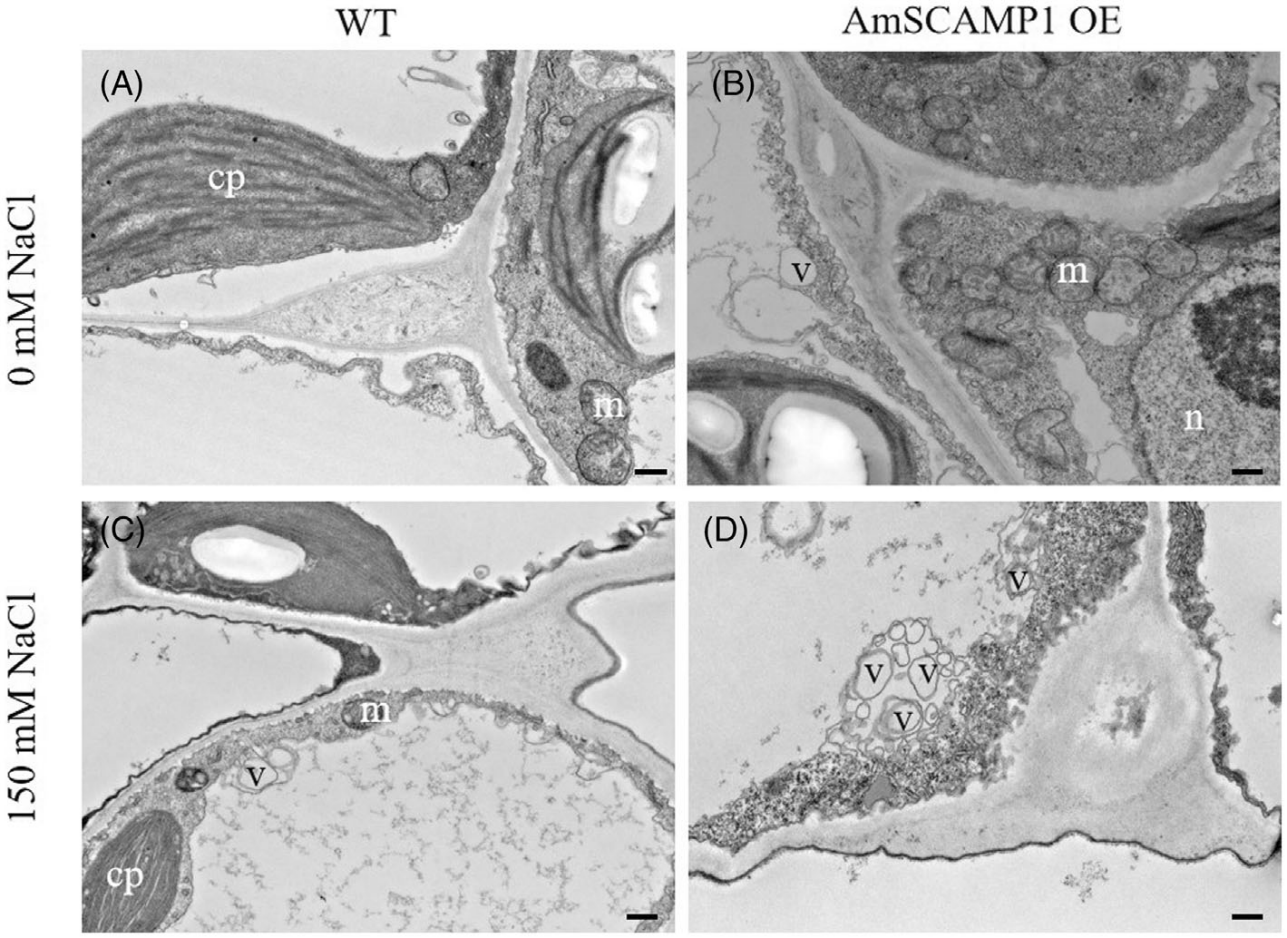
Functional validation of *AmSCAMP1* in transgenic *A. thaliana* using TEM and vesicles observation. *AmSCAMP1*, a vesicle trafficking‐related gene, was transformed into *A. thaliana*; then the vesicles in the transgenic *A. thaliana* leaves treated with 150 mM NaCl were observed under TEM. (A) and (B) show the cellular ultrastructure of wild‐type (WT) and AmSCAMP1 OE under 0 mM NaCl treatment. (C) and (D) show the cellular ultrastructure of wild‐type (WT) and AmSCAMP1 OE under 150 mM NaCl treatment. AmSCAMP1 OE treated with 150 mM NaCl exhibits the most vesicles. cp, chloroplast; m, mitochondria; n, nucleus; v, vesicle. Scale bars of A, B, C, D = 4 μm.

### Analyses of salt gland‐specific transcription factor AmHAT2 binding to the promoters of *AmCLC‐a* and *AmSCAMP1*

To determine whether the three selected genes *AmNHX2*, *AmCLC‐a*, and *AmSCAMP1* are specifically expressed in salt gland, the homologs of the salt gland‐specific HD‐ZIP transcription factor LbHDZ14 from another recretohalophyte *Limonium bicolor* (Wang et al., 2026) were analyzed in the *A. marina* genome. Considering the close evolutionary relationship between LbHDZ14 and the Arabidopsis HD‐ZIP II transcription factor AtHAT2, the homologous protein encoded by Am23029 was identified and designated AmHAT2 (Figure 10A). The promoters of *AmNHX2*, *AmCLC‐a*, and *AmSCAMP1* were analyzed using PlantTFDB (https://plantregmap.gao‐lab.org/binding_site_prediction.php) to identify potential HD‐ZIP binding sites. Putative binding motifs were detected in the promoters of *AmCLC‐a* and AmSCAMP1, but not in *AmNHX2* (Table S4). The predicted motifs were GGCAATCATTG in the *AmCLC‐a* promoter and TCCAATCATTA in the *AmSCAMP1* promoter (Figure 10B,D). AlphaFold3 server was used to predict potential protein–DNA interactions between AmHAT2 and the identified HD‐ZIP binding motifs. The predicted structures showed that the AmHAT2 DNA‐binding domain was positioned directly over the motifs in the promoters of *AmCLC‐a* and *AmSCAMP1*, supporting a potential interaction between AmHAT2 and the target DNA sequences (Figure 10B,D). In contrast, mutation of the binding motifs disrupted the predicted interaction and shifted the DNA‐binding domain away from the original binding interface (Figure 10C,E). To validate these predictions, dual‐luciferase assays were performed in *Nicotiana benthamiana* leaves. Co‐infiltration of 35S::AmHAT2 with the *pAmCLC‐a*::LUC or *pAmSCAMP1*::LUC reporter constructs resulted in markedly stronger luminescence signals compared with the corresponding control treatments (Figure 10F–G). These results suggest that the salt gland‐specific HD‐ZIP transcription factor AmHAT2 binds to the promoters of *AmCLC‐a* and *AmSCAMP1*, potentially contributing to the regulation of their expression in the salt gland of *A. marina*.

**Figure 10:**
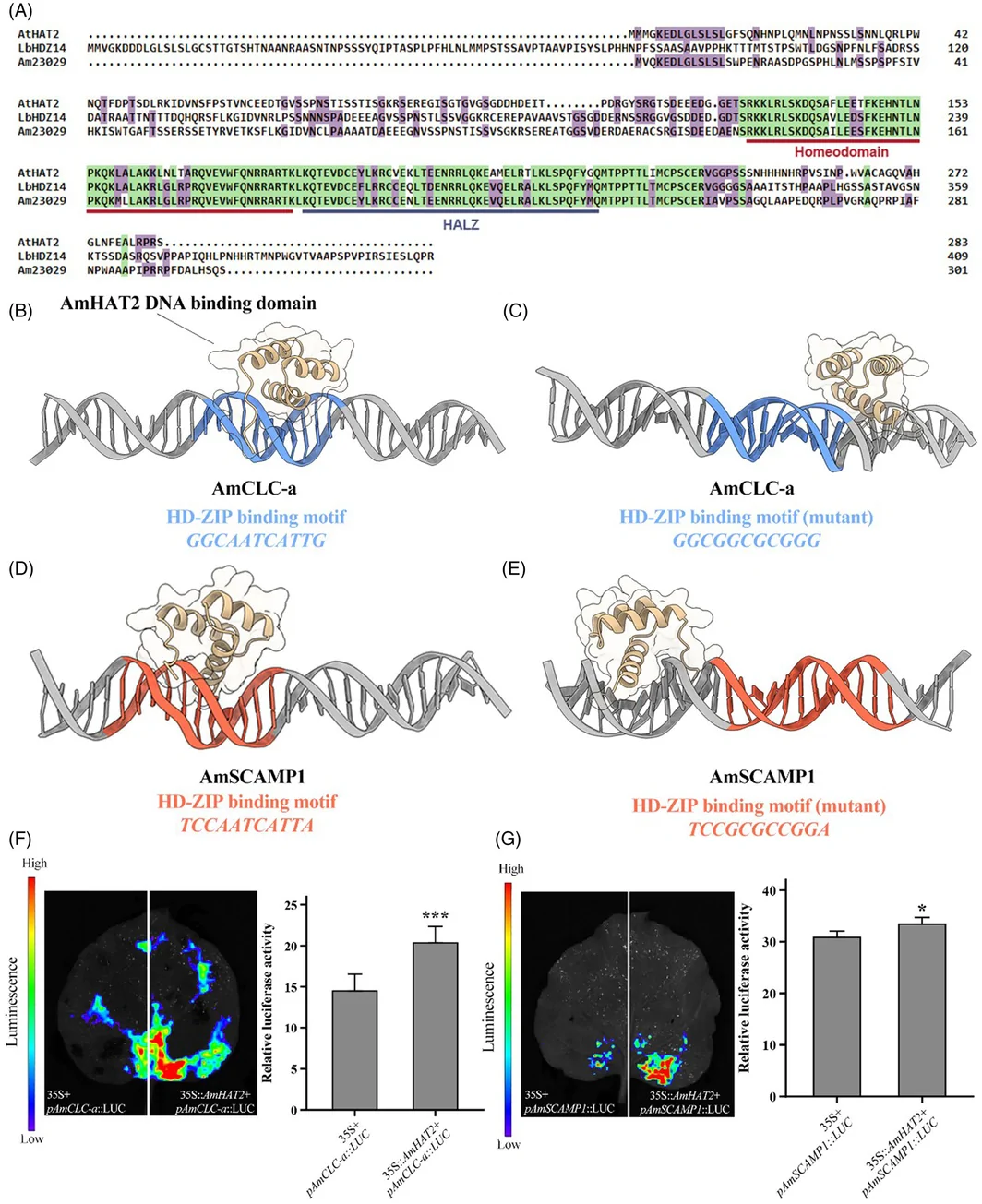
Prediction and validation of AmHAT2 binding to the promoters of *AmCLC‐a* and *AmSCAMP1*. (A) Multiple sequence alignment of AmHAT2, AtHAT2, and LbHDZ14 showing the conserved homeodomain (HD) and homeodomain‐associated leucine zipper (HALZ) regions. (B, C) AlphaFold3‐predicted interactions between the AmHAT2 DNA‐binding domain and the wild‐type (B) or mutated (C) HD‐ZIP binding motif in the *AmCLC‐a* promoter. (D, E) AlphaFold3‐predicted interactions between the AmHAT2 DNA‐binding domain and the wild‐type (D) or mutated (E) HD‐ZIP binding motif in the *AmSCAMP1* promoter. (F, G) LUC assay in *Nicotiana benthamiana* leaves and quantification of luminescence intensity showing the regulation of the *AmCLC‐a* (F) and *AmSCAMP1* (G) promoters by AmHAT2. Data are presented as mean ± SD (*n* = 3). Different letters indicate significant differences (*P* < 0.05).

## DISCUSSION

*A. marina* is a typical salt‐secreting mangrove plant and one of the most widely distributed mangrove species along the tropical and subtropical intertidal zone around the world (Saenger & West, 2018). The function of salt glands is known to exclude Na^+^ and Cl^−^ from leaves; however, the molecular mechanism remains poorly understood. In this study, we observed the Na^+^ and Cl^−^ secretion rate increased with salinity when the salinity was below 400 mM NaCl (Figure 1). In our recent study, we found salinity above 400 mM NaCl inhibits the accumulation of dry matter in *A. marina* (Guo et al., 2023). This observation was also supported by the findings of Chen et al. (2010) who reported that *A. marina* appeared to have the ability to survive under high salinity up to 600 mM NaCl but secrete less salt crystals compared with 400 mM NaCl treatment. Furthermore, Li et al. (2020) reported that the mean salinity in the natural habitat of our sampling site ranges from 12‰ to 26‰. Na^+^ secretions and the expressions of Na^+^ secretion‐related genes were increased when treated salinity was close to seawater, but decreased when treated salinity exceeded seawater in another salt‐secreting mangrove plant *Avicennia officinalis* (Tan et al., 2013). This was further confirmed by the NMT result, in which the maximum Na^+^ and Cl^−^ fluxes were detected in the salt glands of 400 mM NaCl‐treated plants (Figure 1J–M).

The transmission electron microscope showed that the application of 400 mM NaCl increased mitochondria density and vesicles number (Figure 2J–M), which may due to the high requirement for energy and the intense activity of ions transport. The recretohalophytes *Limonium bicolor* and *A. marina* exhibit both similarities and significant differences in their ultrastructure. In both species, the secretory cells of the salt glands are rich in small vesicles and well‐developed mitochondria, lack a central vacuole and chloroplasts, and the salt glands are covered by a cuticle that exhibits strong autofluorescence under ultraviolet excitation (Feng et al., 2015; Figure 2B,C). Study on the structure of the salt gland in *L. bicolor* has found that it is a typical multicellular structure, consisting of 16 cells from five categories: secretory cells, accessory cells, inner cup cells, outer cup cells, and basal cells (Dassanayake & Larkin, 2017). Although the salt glands of *A. marina* are also multicellular, they consist of secretory cells, stalk cell and collecting cells (Figure 2F,G). Furthermore, while the secretory cells of both types of salt glands are covered by a cuticle, the cuticle structure is different. In *L. bicolor*, the cuticle has four secretory pores, which provide direct channels for salt ion excretion (Dassanayake & Larkin, 2017). In *A. marina*, however, the cuticle on top of the secretory cells forms a cuticular chamber that stores salt ions prior to secretion (Figure 2H,I).

To further investigate the salt‐secreting mechanism, we used LCM coupled with RNA‐Seq to characterize the differentially expressed genes (DEGs) in salt glands between the control and 400 mM NaCl‐treated plants. Before laser capture microdissection, we observed the salt glands of epidermal peels after removing the abaxial epidermal cell layers and the mesophyll‐palisade tissues. As Figure 2F,G show, we can obtain the intact salt glands with almost no contamination by other cells. After RNA‐Seq analysis, a total of 1741 DEGs were obtained, of which 1053 were up‐regulated and 688 were down‐regulated (Figure S2; Table S2). Recent studies have shown that the development of salt glands in *L. bicolor* is precisely regulated by a transcriptional regulatory network, with transcription factors such as LbMYB368 and HD‐ZIP positively regulating the salt gland development and salt secretion (Wang et al., 2026; Zhu et al., 2025). However, the LCM‐RNAseq data from our study indicate that under salt treatment, the salt glands of *A. marina* are enriched with functional genes involved in vesicle trafficking, ion transport, compatible solute transport, aquaporins‐based water transport, hormone signaling, cell wall and cuticle wax biosynthesis (Figures 4 and 5).

### LCM‐based transcriptome analysis in *A. marina* salt glands

Laser capture microdissection (LCM) is a technique that enables the identification, selection, and isolation of specific cell populations. Compared with manual dissection under the microscope, LCM offers a more direct approach for isolating plant‐specific tissues or cells. This method allows precise isolation of small numbers of cells, facilitating the study of gene expression profiles in different tissues or cells without the enzymatic digestion procedure or cell‐specific reporter gene (Qin et al., 2022; Saint‐Marcoux et al., 2015). In *Artemisia annua*, glandular trichomes were isolated from leaf and flower bud sections fixed with 4% paraformaldehyde using LCM (Olsson et al., 2009). In *Citrus reticulata*, nucellar and integument tissues were obtained by LCM following fixation with Carnoy's fixative (Lu et al., 2023). Although studies have shown that salt gland cells can be isolated from the upper epidermis of *A. marina* following enzymatic digestion, the epidermal peeling process involves enzyme treatment that may compromise the integrity of RNA in salt gland cells (Tan et al., 2010). In this study, LCM was innovatively applied to isolate salt gland cells from *A. marina* for subsequent transcriptomic analysis. Unlike previous transcriptomic studies using whole leaves, the precise isolation of salt gland cells by LCM avoids transcriptional interference from mesophyll and epidermal cells, enabling a more accurate dissection of the salt‐responsive gene expression profiles in salt glands. Following the protocol of Lu et al. (2023), we firstly fixed the upper epidermal cells of *A. marina* leaves by vacuum infiltration with Carnoy's fixative to inactivate various enzymes and biochemical reactions. Subsequently, a magnification of 40× was used to ensure precise cutting of salt gland cells. In addition, the entire LCM program is controlled within 1.5 h.

### Ion transport

It was found that there exist noticeable ion gradients across the vacuole in the cytoplasm of the salt glands (Zou et al., 2017). In this process, H^+^‐ATPase generates proton gradients across the plasma and vacuolar membranes (Su et al., 2019). Our data showed that genes encoding plasma membrane H^+^‐ATPase, tonoplast membrane H^+^‐ATPase subunit, and phosphorylated proton pump were up‐regulated in the salt glands of salt‐treated plants (Figure 4A). Furthermore, it showed an increase in mitochondrial density in the salt glands from salt‐treated plants (Figure 2L,M). In addition, the transcript levels of five sugar transporters were also up‐regulated (Figure 4D). Considering the lack of chloroplasts in the salt glands, they may be involved in transporting sugar into the salt glands to support the energy needs.

We also identified 23 genes encoding ion transporters in the salt glands, including the anion transporter, nitrate transporter (*NRT*) and chloride channel (*CLC*), the cation transporters, Na^+^/H^+^ antiporter (*SOS1/NHX*), potassium transporter (*AKT*), and cyclic nucleotide‐gated ion channel (*CNGC*) (Figure 4A).

NRTs and CLCs were involved in Cl^−^ transport in plants. For example, AtCLC protects cytoplasm from reaching toxic levels of Cl^−^ by sequestrating them into vacuoles (Jossier et al., 2010). The nitrate transporter NRT1.1 was also shown to have Cl^−^ permeability in Arabidopsis (Liu et al., 2020). There is ample evidence that Na^+^/H^+^ antiporter and HKT1 systems are major players in plant Na^+^ transport across the membrane (Zou et al., 2017). A recent study illustrated that the Na^+^/H^+^ antiporter is critical for salt secretion from the salt glands in the halophyte *Karelinia caspia* under saline conditions (Guo et al., 2020). In addition, NHXs mediate Na^+^ compartmentalization in vacuoles (Tang et al., 2020). HKT can mediate voltage‐dependent Na^+^ influx into salt bladders in *Chenopodium quinoa* (Bohm et al., 2018). Cyclic nucleotide‐gated channel (CNGC) is a non‐selective ligand cation‐gated channel located in the membrane. The expression of CNGC in heterologous systems indicated that plant CNGC has permeability to monovalent and divalent cations (Maser et al., 2002). In the present study, the expressions of these genes showed significant alterations in response to salt treatment (Figures 3A and 4A). Collectively, the DEGs related to ion transport suggested that Na^+^ and Cl^−^ are expelled from mesophyll through SOS1 and CLCs, respectively, and then loaded into salt gland cells through HKT, AKT, NRT, and CNGC, where they are sequestered in the vesicles through NHXs and CLCs or excreted through SOS1 and CLCs. These transport processes are energized by plasma and tonoplast H^+^‐ATPase.

To validate the functional roles of salt ions transport related DEGs, we performed the substrate sensitivity assays using transgenic yeast cells transformed by the identified genes including *AmNHX2* and *AmCLC‐a* (Figure 6A). *AmNHX2* belongs to the Na^+^/H^+^ antiporter family. AtNHX1 has been shown to localize to the tonoplast and function in sequestering Na^+^ into the vacuole in Arabidopsis (Sottosanto et al., 2004). In our study, the expression of *AmNHX2*, which is homologous to *AtNHX1*, was significantly up‐regulated in the salt glands (Figure 4A), and the expression in yeast (both BY4741 line and AXT3K line) confirmed that it enhances salt tolerance in transgenic yeast (Figure 6A,E). In addition, the salt ions accumulation assays using above transgenic yeast cells transformed by *AmNHX2* and *AmCLC‐a* (Figure 6), and tobacco leaves overexpressing *AmNHX2* and *AmCLC‐a* (Figure 7) demonstrated that those two DEGs can change the contents of Na^+^ and Cl^−^ in the transgenic yeast cells or transgenic tobacco leaves under salt treatments, implying those genes do play salt ions transport functions.

### Vesicle trafficking

The hypothesis has been proposed that vesicle trafficking participates in salt secretion from salt glands in recretohalophytes (Barhoumi et al., 2008; Yuan et al., 2015). In this study, we found a large number of vesicles in the secretory cells. Moreover, the number of vesicles in the secretory cells increased significantly after salt treatment (Figure 2J,K). In addition, 13 genes related to vesicle formation (*SAR1A*, *ALA3*), vesicle transport (*ECHIDNA*, *VSRs*), and membrane fusion (*SYPs*, *SYT*, *SCAMPs*) were identified (Figure 4B).

GTP‐binding protein (SAR1) functions as a switch to control the assembly of coat protein complex II (COPII) that directs vesicle budding from ER. It has been shown that overexpression of *AtSar1a* in Arabidopsis enhances its secretion activity from ER membranes to cytosol (Memon, 2004). Aminophospholipid ATPases (ALAs) are involved in flipping specific lipids, producing membrane curvature and assisting vesicle formation (Poulsen et al., 2008). In our study, the transcriptional levels of *SAR1A* and *ALA3* increased in the salt glands of 400 mM NaCl‐treated plants. This may be beneficial to the production of more vesicles in the salt glands of *A. marina*. Golgi apparatus membrane protein‐like protein (ECHIDNA) is involved in protein transport from ER to Golgi apparatus. Compared with wild‐type, the Arabidopsis ECHIDNA mutant exhibits defects in trans‐Golgi‐network (TGN) structure and function, manifested by mis‐localization of several TGN‐localized proteins including vacuolar H^+^‐ATPase subunit a1 (VHA‐a1) (Gendre et al., 2011). Vacuolar‐sorting receptors (VSRs) are considered to recognize cargo proteins at the TGN (Niemes et al., 2010). In addition, V‐ATPase and NHXs not only participate in the compartmentalization of Na^+^ in vacuoles but also transport vesicles to different regions (Bassil et al., 2011). When vesicles are transported near the cell membrane, they fuse with the cell membrane under the participation of different proteins and then the cargos are released. For example, exocyst complex component (EXO70A1) participates in binding vesicles to the cell membrane (Vukasinovic et al., 2017). Soluble N‐ethyl‐maleimide sensitive factor attachment protein receptor (SNARE) cooperates with synaptotagmin (SYT) to accelerate fusion of vesicles membrane to cell membrane by binding simultaneously to the two membranes (Schapire et al., 2008). In addition, secretory carrier membrane protein (SCAMP) regulates exocytosis of exocrine gland cells (Law et al., 2012). In plants, overexpression of *SCAMP1* in transgenic tobacco BY‐2 cells resulted in the transfer of SCAMP1 localization from the cytoplasm to the cell membrane (Lam et al., 2009). In our study, the increased expression of *EXO70A*, *SYTs* and *SCAMP* may contribute to the fusion of vesicles to plasma membrane.

AmSCAMP1 is a member of the secretory carrier membrane protein (SCAMP) family. SCAMP proteins have been reported to participate in vesicle trafficking in both plants (Law et al., 2012) and animals (Maruyama et al., 2026). In our study, *AmSCAMP1* was significantly up‐regulated under salt treatment. To validate the functional roles of vesicle formation and trafficking related DEGs in salt tolerance, we firstly focused on *AmSCAMP1* to conduct the substrate sensitivity assay using transgenic yeast cells. The result showed that the yeast transformed with *AmSCAMP1* grew significantly better than the pYES2 empty vector under 500 mM NaCl treatment (Figure 6B). Furthermore, overexpression of *AmSCAMP1* in Arabidopsis led to a significant increase in vesicle number under salt stress (Figure 9D), which is consistent with the function of AtSCAMP1 in regulating exocytosis in tobacco BY‐2 cells (Lam et al., 2009). We further verified the function of *AmSCAMP1* in salt tolerance using *AmSCAMP1*‐overexpressing Arabidopsis. The results showed that under salt treatment, the root length and fresh weight of OE2 and OE3 were higher than those of the wild‐type (Figure 8A–C), indicating that *AmSCAMP1* can promote Arabidopsis growth under salt treatment. In addition, the Na^+^ content in OE2 and OE3 was lower than those in wild‐type plants (Figure 8F), suggesting that *AmSCAMP1* contributes to improving plant salt tolerance. To elucidate the underlying mechanism, we further analyzed the Na^+^ flux at the root tips of transgenic Arabidopsis using NMT and found that compared with wild‐type plants, the transgenic lines exhibited Na^+^ efflux under salt treatment (Figure 8D,E). Moreover, the expression level of *AtSOS1* in OE2 and OE3 was significantly higher than that in the wild‐type (Figure 8G), indicating that overexpression of *AmSCAMP1* in Arabidopsis could facilitate Na^+^ homeostasis. In summary, the results of this study provide strong evidence for the function of *AmSCAMP1* in salt secretion by salt glands of mangrove plant *A. marina*, and reveal its important role in plant salt tolerance.

Based on the above results, we speculate that salt treatment can promote the secretion of Na^+^ by increasing the vesicles budding (*SAR1A, ALA3*), vesicles transport from ER to Golgi apparatus (*ECHIDNA*, *VSRs*, *V‐ATPase*, *NHXs*), and the fusion of vesicles to plasma membrane (*EXO70A1*, *SYTs*, *SCAMPs*) in the salt glands of *A. marina*.

### Aquaporin‐based water transport

Aquaporins (AQPs) belong to a superfamily of major intrinsic proteins (MIPs), which play a role in regulating transcellular water flow (Vera‐Estrella et al., 2004). Studies have shown that AQPs are involved in the salt secretion of salt glands (Tan et al., 2013). Two classes of aquaporin genes encoding plasma membrane intrinsic proteins (PIPs) and tonoplast intrinsic proteins (TIPs) were preferentially expressed in the salt gland cells, and they were rapidly induced in response to increasing salt concentration (Tan et al., 2013). In this study, we identified 14 aquaporin genes in the salt glands, most of which were up‐regulated under salt treatment (Figure 4C). According to a previous study and our transcriptome data, we proposed that the role of aquaporins in salt secretion by salt glands includes excretion of water to carry away salt and osmotic regulation.

### Compatible solutes transport

Accumulation of compatible solutes is considered a key adaptation mechanism in plants' response to abiotic stress (Lehmann et al., 2010). For example, Na^+^ accumulating in the vacuoles of salt bladder in salt‐treated *M. crystallinum* are balanced by pinitol and proline in the cytosol (Oh et al., 2015). Plant compatible solutes are classified into three major groups including amino acids, polyol and/or sugars, and quaternary amines (Bohm et al., 2018). In this study, amino acids transporter genes *ProT1*, *AAPs*, and *LHTs* were significantly up‐regulated in the salt glands under salt treatment (Figure 4D). Besides, we also found that sucrose transporter and polyol transporter increased after salt treatment (Figure 4D). Above results suggest these genes involving in the production of a wide variety of organic compatible solutes play a vital role for the salt secretion from the leaf salt glands of *A. marina* under salt treatment.

On the other hand, the intercellular transport of macromolecules including compatible solutes can also efficiently pass through plasmodesmata (Zhang et al., 2021). Studies have shown that the regulation of plasmodesmata aperture is mediated by the synthesis/degradation of the (1,3)‐β‐glucan polymer callose (Amsbury et al., 2018). In this study, the plasmodesmata between the stalk cell and the secretory cells are more abundant in NaCl‐treated plants compared with the CK plants (Figure 2N–Q). Their high abundance may signify the intense transport of materials between the stalk cell and the secretory cells. More importantly, the genes encoding glucan endo‐1,3‐beta‐glucosidase were significantly up‐regulated in the salt glands under salt treatment (Figure 5C). It is reported that this enzyme is primarily expressed in epidermal cells and plays a role in degrading (1,3)‐β‐glucan polymer callose around the plasmodesmata (Zavaliev et al., 2011). Therefore, it is reasonable to speculate that the up‐regulation of glucan endo‐1,3‐beta‐glucosidase gene in the salt glands contributes to the intense transport of macromolecules and small molecules between the stalk cell and the secretory cells, thereafter further promoting the process of salt secretion.

Overall, our RNA‐Seq results suggest salt‐induced expression of genes related to compatible solutes transport is very important in maintaining the osmotic potentials during the salt secretion from the salt glands of *A. marina*.

### Abscisic acid and ethylene signaling transduction

In this study, we obtained the DEGs related to abscisic acid (ABA) and ethylene (ETH) signal transduction (Figure 5A,B). For ABA signaling, six genes encoding two 9‐cis‐epoxycarotenoid dioxygenase1 (*NCED1*), two abscisic acid receptor 4 (*PYL4*), and two abscisic acid 8′‐hydroxylase (*CYP707A*) were identified (Figure 5A). For ETH signaling, one gene encoding ethylene receptor 1 (*ETR1*) and ten genes encoding ethylene responsive transcription factors *AP2/ERF* were identified in the salt glands of *A. marina* (Figure 5B).

NCED catalyzes the oxidative cleavage of 9‐cis‐epoxycarotenoid, which is considered as the critical rate‐limiting step in ABA biosynthesis in higher plants (Nakashima & Yamaguchi‐Shinozaki, 2013). PYLs are reported as ABA receptors that bind to ABA and perform ABA signal transduction in response to a variety of abiotic stresses (Zhang et al., 2017). In contrast to ABA biosynthesis, the key step of ABA deactivation is the hydroxylation of the 8′‐methyl group of ABA, which is catalyzed by ABA 8′‐hydroxylase (CYP707A) to yield 8′‐hydroxy‐ABA (8′‐OH‐ABA) (Saito et al., 2004). It has been reported that ABA negatively regulates ABA accumulation by enhancing the ABA 8′‐hydroxylase level, and the transcript levels of *CYP707A* genes increased in response to ABA (Saito et al., 2004). In this study, our results showed that salt treatment significantly decreased the transcriptional levels of the ABA biosynthesis gene *NCED1* and the ABA receptor gene *PYL4*, while significantly increasing the transcriptional levels of the ABA catabolism related gene *CYP707A* in the salt glands of *A. marina* (Figure 5A). These data suggest that the ABA level fine‐tuning by the ABA biosynthesis, ABA catabolism and ABA perception plays an essential role in salt secretion from the salt glands of *A. marina* leaves.

It is well‐known that ethylene (ETH), a plant hormone, plays a critical role in plant development and response to stress (Binder, 2020). In this study, we identified one ethylene receptor 1 gene (*ETR1*) and ten ethylene responsive transcription factor genes *AP2/ERF* in the salt glands of *A. marina* (Figure 5B). In the absence of ethylene, the ETR1 is in an activated state, which can activate the function of CONSTITUTIVE TRIPLE RESPONSE 1 (CTR1), that is, a negative regulator in ETH signaling (Bakshi et al., 2015). Ethylene responsive factor AP2/ERF can mediate plants' abiotic stress by regulating the expression of downstream genes (Zhang et al., 2012). For example, they revealed that ERF98 can improve the salt tolerance of Arabidopsis by enhancing the ascorbic acid (AsA) biosynthesis‐related gene *VTC1*. In the present study, the expression of the ETH receptor *ETR1* and half of the ethylene responsive transcription factors *AP2/ERF* in the salt gland cells was mostly up‐regulated under salt treatment, indicating that the ethylene signaling pathway is involved in the salt secretion process from the salt glands in *A. marina* leaves under salt treatment.

### Cell wall and cuticle wax biosynthesis

The plant cell wall is a dynamic network structure composed of cellulose microfibrils and spiral cellulose embedded in a pectin matrix (Wang et al., 2016). Cellulose synthase‐like (CSL) genes are predicted to catalyze the biosynthesis of non‐cellulosic polysaccharides such as the β‐D‐glycan backbone of hemicelluloses (Yoshikawa et al., 2013). Galacturonosyltransferase (GAUT) is among the enzymes required for the biosynthesis of the plant cell wall pectin (Fabiana et al., 2013). In addition, pectin methylesterases catalyze the demethylesterification of cell wall polygalacturonans and its activity is specifically regulated by pectin methylesterase inhibitors (PMEIs) (Li et al., 2022). In the present study, the up‐regulation of genes encoding cellulose synthase‐like protein (*CSLs*) and galacturonosyltransferase (*GAUT*), and the down‐regulation of genes encoding pectin methylesterase inhibitors may lead to the increased biosynthesis of cellulose and pectin (Figure 5C). Cell wall formation is positively regulated by a variety of proteins, such as receptor‐like protein kinase (FERONIA), fascilin‐like arabinogalactan protein (FLA), as well as G‐type lectin S‐receptor‐like serine/threonine‐protein kinase (RLK). In this study, the expressions of *FERONIA*, *FLA* and *RLK*, the genes encoding the above proteins, showed to be enhanced in the salt glands under salt treatment (Figure 5C). Therefore, our data suggest that salt treatment can enhance the biosynthesis and deposition of cell wall polymers in the salt glands by up‐regulating the genes related to cell wall biosynthesis.

The ductility of the cell wall is an important factor in maintaining normal turgor pressure under salt stress (Ye et al., 2008). In the present study, salt treatment increased the expression of several genes associated with cell wall extensibility, such as xyloglucan endotransglucosylase/hydrolase protein (*XTH*), expansin like proteins (EXPs), and glucan endo‐1,3‐beta‐glucosidase (*Glu*), implying that salt treatment is likely to increase the cell wall extensibility of salt glands and help them to regain turgor pressure in response to elevated salt concentrations in the salt glands.

In this study, transmission electron microscopy revealed that the cuticle on the upside region of the secretory cells is separated from the cell wall, and a subcuticular collecting cavity is formed (Figure 2H,I). In addition, Oi et al. (2013) found ions are accumulated in this cavity and the increased hydrostatic pressure allows the solution to permeate easily through the cuticle from the cavity to the outside of the salt gland (Oi et al., 2013). Further observation showed that the bicellular glands of *C. gayana* excrete salts just above their cap cells without rupturing the cuticle. They explained that the lack of cuticular wax on the cap cells implies that the cuticle on cap cells is more permeable than on other epidermal cells and therefore allows the solution to move more easily out of the cap cells (Oi et al., 2014).

Interestingly, these genes related to cuticle wax biosynthesis and transport were down‐regulated in the salt glands of salt‐treated *A. marina* plants in the present study (Figure 5D). For example, 3‐ketoacyl‐CoA synthase (KCS) is the rate‐limiting enzyme in the biosynthesis of very long‐chain fatty acids (VLCFA) (Yang et al., 2021). ECERIFERUM (CER) is involved in all VLCFA elongation reactions in plants and the absence of CER activity results in a reduction of cuticular wax formation (Yang et al., 2020). In addition, long‐chain acyl‐CoA synthetase (LACS) is also involved in the biosynthesis of waxes in the cuticle. In the *lacs1* mutant of Arabidopsis, the epidermal fatty acid content of leaves was reduced by 22% (Lu et al., 2009). ABCG11 is involved in the wax transport from the plasma membrane to the extracellular space (Lee & Suh, 2013). Lipid transfer proteins (LTPs) assist the waxes to cross the plant cell wall, while knockdown of lipid transfer protein GPI‐anchored 2 (LTPG) significantly reduced the alkane content in Arabidopsis epidermal waxes (Kim et al., 2012). This study found that genes related to wax synthesis in the cuticle layer (such as *KCS*, *CER26*, *ABCG11*) were significantly down‐regulated under salt treatment (Figure 5D). This contrasts with most salt stress studies, where wax‐related genes are commonly reported to be up‐regulated (Lee & Suh, 2013; Luo et al., 2024; Yang et al., 2025). We speculate that the salt gland of *A. marina* may have a unique strategy to increase the permeability of the cuticle layer by downregulating wax synthesis, thereby facilitating the excretion of salt solution from secreting cells. This may represent a special mechanism of cuticle‐regulated salt secretion in *A. marina*.

In this study, we used laser capture microdissection coupled with RNA‐Seq to present the tissue specific roles of the salt glands in salt adaptation of mangrove plant *A. marina*. The results demonstrate that ion transport, vesicle trafficking, aquaporin‐based water transport, compatible solutes transport, ABA and ethylene signaling, cell wall and cuticle wax biosynthesis and transport were significantly altered under salt treatment, implying these processes may play an important role in salt secretion from the salt glands of *A. marina*. Based on these findings and the report of Ramakrishna et al. (2025), we drew the proposed working model of salt secretion in the salt glands of *A. marina* (Figure 11).

**Figure 11:**
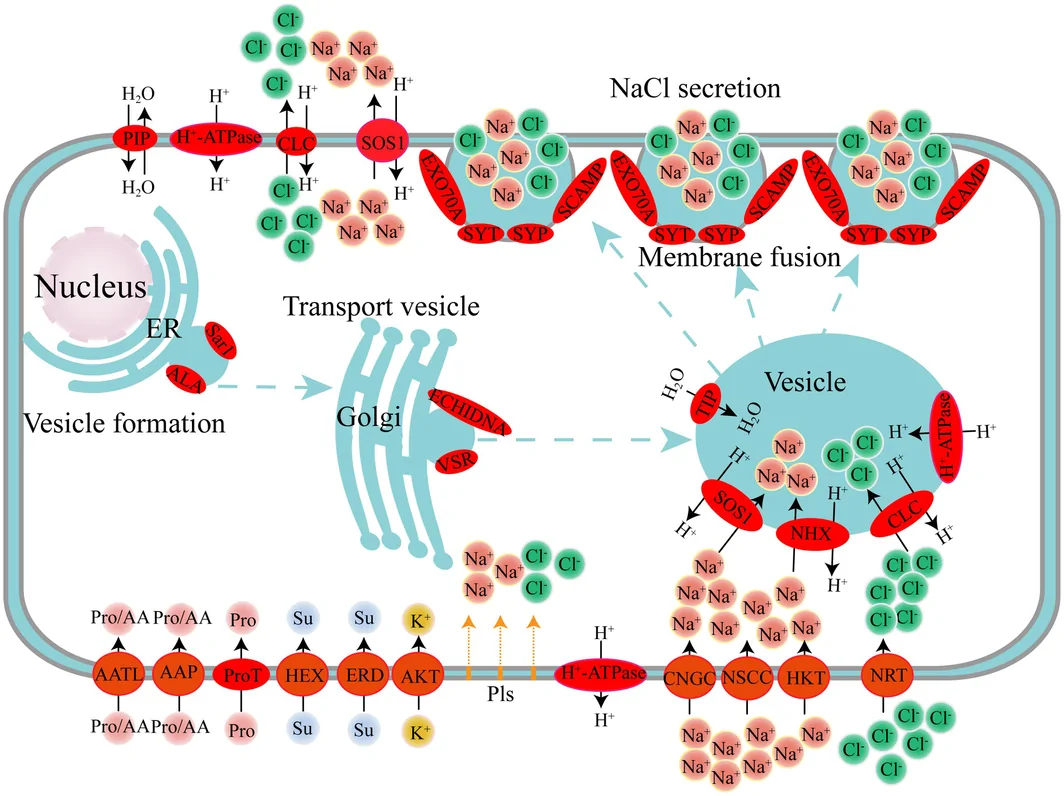
Schematic model for salt secretion through vesicle trafficking in *A. marina* salt glands. AA, Amino acid; AAP, Amino acid permease; AATL, Proline and amino acid permease; AKT, Potassium transporter; ALA, Aminophospholipid ATPases; CC, Collecting cell; CLC, Chloride channel protein; CNGC, Cyclic nucleotide‐gated ion channel; ECHIDNA, Golgi apparatus membrane protein‐like protein; ER, Endoplasmic reticulum; ERD, Sugar transporter like protein; EXO70A1, Exocyst complex component; HEX, Hexose carrier protein; HKT, High‐affinity K^+^ transporter; H^+^‐ATPase, Plasma membrane ATPase; NHX, Na^+^/H^+^ antiporter; NSCC, Non‐selective cation channels; NRT, Nitrate transporter; PIP, Plasma membrane aquaporin; Pls, Plasmodesmata; Pro, Proline; ProT, Proline transporter; Sar1, GTP‐binding protein 1; SC, Secreting cell; SCAMP, Secretory carrier‐associated membrane protein; SOS1, Na^+^/H^+^ antiporter; ST, Stalk cell; Su, Sugar; SYP, Syntaxin; SYT, Synaptotagmin; TIP, Tonoplast aquaporin; VSR, Vacuolar‐sorting receptor.

## MATERIALS AND METHODS

### Plant materials and treatments

*Avicennia marina* (Forsk.) Vierh. fruits were collected from Zhangjiang River Estuary Mangrove National Natural Reserve (23°55′ N, 117°26′ E). Healthy fruits were planted in pots (40 cm × 30 cm × 30 cm) filled with clean sands. Plants were grown in an environmentally controlled greenhouse at 60–70% relative humidity and around 26 ± 2°C temperature with a day/night cycle of 12 h/12 h by photosynthetically active radiation of 800–1000 μmol m^−2^ s^−1^ in Hoagland's nutrient solution (Li et al., 2021). The Hoagland nutrient solution was applied twice a week and always maintained in the pots. Plants were raised 2 months before the initiation of salt treatment.

*A. marina* seedlings with similar size and uniform growth were selected and treated with Hoagland nutrient solution containing 0, 100, 200, 400, and 600 mM NaCl, respectively, for the follow‐up experiments, with at least three replicates of 15–20 plants per treatment. As to the 200–600 mM NaCl treatment groups, NaCl was added with 100 mM every 3 days until the required salinity. The treatment solutions were renewed every 3 days. After 30 days of salt treatment, some plants were used for physiological measurements, NMT and electron microscopy observation, while others were used for LCM‐RNAseq analysis.

### Observation and analysis of salt secretions

After 30 d of salt treatment, the second paired leaves from the top were used to observe salt crystals. Briefly, the harvested leaves after salt treatment were cut into segments. Then, segments were mounted on a sample stub. The salt crystals were photographed with a stereomicroscope (M165FC, Leica).

The salt crystals were further analyzed with scanning electron microscope (SEM) coupled with energy dispersion analysis X‐ray (EDAX) (Tan et al., 2013). Briefly, the salt crystals from the adaxial epidermis were carefully collected with a brush and then were mounted on the sample stub. Finally, the salt crystals were observed using SEM equipped with EDAX with an accelerating voltage of 15 kV (supra 55 sapphire, Carl Zeiss). The images and records of EDAX spectra were collected and further analyzed for the relative contents of ions.

### Measurements of Na^+^ and Cl^−^ content in the salt gland secretions and the leaves

The Na^+^ and Cl^−^ contents in salt gland secretions of *A. marina* leaves were determined according to the method described by Chen et al. (2010). The adaxial surfaces of the leaves were rinsed with 20 mL of Milli‐Q water to collect the secretions. The collected secretions were filtered with a 0.45 μm microporous membrane. The Na^+^ and Cl^−^ content was measured with ICP‐MS (7500ce, Agilent Technologies, Santa Clara, CA, USA) and ion chromatography (ICS‐5000, Thermo Fisher Scientific, Waltham, MA, USA), respectively.

The Na^+^ and Cl^−^ content in the leaves was measured according to the previous report (Tan et al., 2013). Briefly, the leaves were dried to constant weight and ground with mortar. Then, the samples (1 g) were mixed with nitric acid (8 mL) and digested using a microwave digestion machine (MARS6, CEM). Then, the digestion solution was diluted to 50 mL with distilled water accurately. The Na^+^ and Cl^−^ content was measured with ICP‐MS (7500ce, Agilent) and ion chromatography (ICS‐5000, Thermo), respectively.

### Measurement of net Na^+^ and Cl^−^ flux from the salt glands

Plant leaves were washed three times with Milli‐Q water to remove surface ions and quickly dried on both sides with absorbent paper. The adaxial epidermis of *A. marina* leaves was carefully peeled off and cut into 0.2 cm × 0.2 cm pieces, which were immediately incubated in the measuring solution (0.1 mM KCl, 0.1 mM CaCl_2_, 0.1 mM MgCl_2_, 0.5 mM NaCl, 0.2 mM Na_2_SO_4_, 0.3 mM MES, pH 6.5) for 2 h according to the previous study (Chen et al., 2010). Then, the adaxial epidermis was transferred to the bottom of the measuring chamber containing 5–10 mL of the fresh measuring solution.

Net Na^+^ and Cl^−^ fluxes from the salt glands of *A. marina* leaves were measured using NMT as described by Chen et al. (2010). The microelectrode was first salted with tributyl chlorosilane and the tip was filled with a specific ion exchanger. The plant sample was fixed on the bottom of the measuring chamber filled with measuring solution, followed by the microelectrode mounted on a 3D micromanipulator, and the electrode tip was placed 30 μm above the salt gland. The electrochemical potential difference between the two locations of each electrode was recorded by ASET 2.0 (Sciencewares, Falmouth, MA 02540, USA) software and converted to ion concentration difference using the calibrated slope. Net ion fluxes were calculated using imFluxes 2.0 (Younger USA LLC, Amherst, MA 01002, USA). Data were collected every 6 s, and each point represented the average of 6 samples.

### Transmission electron microscopy

The transmission electron microscopy (TEM) samples were prepared according to De Vargas et al. (2019). The leaves of *A. marina* were washed with Milli‐Q water and cut into small pieces of 2 mm × 2 mm. The segments were immersed in 2.5% glutaraldehyde solution (pH 7.4) and placed in a refrigerator at 4°C overnight. The samples were rinsed 3 times with 0.1 M phosphate acid buffer (pH 7.0) for 15 min each time. The samples were fixed with 1% osmic acid for 2 h. Then, the samples were rinsed with 0.1 M phosphoric acid buffer (pH 7.0) 3 times, each time for 15 min. Then, the samples were dehydrated with ethanol acetone gradient and embedded with Spurr's epoxy resin. The samples were sliced with an ultramicrotome (UC7, Leica) with a thickness of 70–90 nm and stained with uranyl acetate lead citrate. Finally, the samples were observed with a TEM (HT‐7800, Hitachi).

### Collection of salt glands by laser capture microdissection

The adaxial peels were obtained with a razor blade to remove abaxial epidermal layers and mesophyll cells of leaves of *A. marina* according to Tan et al. (2010) and Chen et al. (2010). Then, the adaxial peels were cut into segments (1 cm × 1 cm). The adaxial peels floated on at least 20 volumes of freshly prepared 3:1 ethanol: acetic acid were vacuum infiltrated for 10 min and fixed at 4°C for 8 to 10 h (Zhan et al., 2015). Then, the samples were removed from fixative and placed on RNase‐free polyethylene naphthalate (PEN) membrane slides for processing. The entire process from leaf collection, epidermal peeling to laser cutting is rapidly completed under low‐temperature conditions to maximize the inhibition of endogenous RNase activity. All tools and solutions used were commercially available nuclease‐free products. The entire LCM program is controlled within 1.5 h.

Laser capture microdissection (LCM) was performed using Leica LMD7 microdissection systems as described by Kerk et al. (2003). Load PEN slides onto the microscope and a 0.5 ml RNase‐free tube was placed in a collector so that the lid of the cap was just beneath the salt glands. Salt glands were observed with a 40× objective and selected using the graphics tools of the Leica LDM Software and were cut with a UV laser through the microscope lens. Captures were performed using 10 beam diameters at a power setting of 50 mW, and laser pulse durations were 650 μs and 2.5 ms to obtain a clean, narrow excision of salt glands. Salt glands from adaxial peels were dropped into the lid of a 0.5 ml RNase‐free tube filled with 20 μL lysis solution from Rneasy Micro kit (Qiagen). Typically, the total area of LCM‐isolated epidermis enriched by salt glands was at least 12 mm^2^. At the end of every session (usually 1 h), the collecting tube was snap‐frozen in liquid nitrogen. All the dissected samples were stored at −80°C until RNA isolation.

### RNA extraction, transcriptome sequencing and data analysis

RNA from LCM salt gland cells was extracted using the Rneasy Micro kit (Qiagen) according to the manufacturer's instructions. Total RNA quality was assessed using the RNA Pico Assay for 2100 Bioanalyzer. For LCM‐derived samples, approximately 12–20 mm^2^ of LCM‐collected tissues typically yielded 15 to 25 ng of total RNA. Then the low input total RNA library was constructed by using SMART seq v4 kit lib (Takara Inc.). Three biological repetitions were used per sample.

Transcriptome sequencing was performed on the Illumina HiSeq platform. Clean reads were generated by filtering adapter‐containing reads, reads containing more than 10% ambiguous bases (‘N’) and reads with more than 50% of bases showed a Qpred ≤20. Then the clean reads were aligned to the *A. marina* reference genome built by our laboratory (https://jasonxu.shinyapps.io/MangroveDB/) using HISAT software. The gene expression levels were analyzed using RPKM method (fragments per kilobase of transcript per million fragments mapped). The DESeq2 R package were used to perform differentially expressed genes (DEGs) analysis (Love et al., 2014; Trapnell et al., 2010). Genes with the threshold of the adjusted *P* value (*P*adj) < 0.05 and |log2(FoldChange)| > 1 were defined as DEGs. Gene ontology (GO) enrichment analysis of DEGs was performed with GOseq R package. GO terms with corrected *P*‐values <0.05 were defined as significantly enriched and used to identify the primary biological process of the DEGs. Visual analysis of GO terms enriched in DEGs were analyzed using BINGO (Maere et al., 2005).

### Salt tolerance assay using transgenic yeast cells

The DNA fragments of *AmNHX2*, *AmCLC‐a*, and *AmSCAMP1* genes were inserted into the yeast (*Saccharomyces cerevisiae*) expression vector pYES2:GFP using the ClonExpress® II One Step Cloning Kit (Vazyme, China), resulting in the construction of pYES2:AmNHX2‐GFP, pYES2:AmCLC‐a‐GFP, and pYES2:AmSCAMP1‐GFP vectors. The primers used in the experiments are listed in Table S3. The empty vector pYES2:GFP served as the control. The recombinant plasmids pYES2:AmNHX2‐GFP, pYES2:AmCLC‐a‐GFP, and pYES2:AmSCAMP1‐GFP, along with the control vector, were transformed into the yeast strain BY4741 using the lithium acetate method (Zhang et al., 2023).

Positive clones were selected on SD‐Ura medium containing 2% (w/v) glucose. For sensitivity analysis, recombinant yeast cells stored in SD‐Ura medium were activated and adjusted to an OD600 of 1. A series of five 10‐fold dilutions were prepared, and 5 μL of each dilution was spotted onto solid SD‐Ura medium containing 2% (w/v) glucose (as the control) or galactose supplemented with 500 mM NaCl. Subsequently, the plates were incubated at 29°C for 6 days, and the growth results were documented using a camera. After cultivation, yeast cells were collected by centrifugation at 4000 rpm and 4°C, and washed three times with sterile water. The collected yeast cells were dried in an oven and the dry weight was weighed. After the yeasts were completely digested with concentrated nitric acid, the contents of Na^+^ and Cl^−^ in the transgenic yeast (BY4741) transformed by pYES2:AmNHX2‐GFP and pYES2:AmCLC‐a‐GFP were measured using ICP‐MS (7500ce, Agilent) and ion chromatography (ICS‐5000, Thermo), respectively.

Next, using Na^+^ transport deficient yeasts (AXT3K line), the role of *AmNHX2* in Na^+^ transport was further analyzed. The experimental procedures and yeast cultures were the same as above‐mentioned. The growth patterns of empty and transgenic yeasts (AXT3K line) on the glucose plate and on the galactose plate with 15 mM NaCl were recorded. Finally, the contents of Na^+^ in the empty and AXT3K yeasts cells transformed by *AmNHX2* under 15 mM NaCl were measured by the ICP‐MS method.

### Na^+^ and Cl^−^ accumulation assay, subcellular localization analysis of AmNHX2 and AmCLC‐a using transgenic tobacco leaves

The coding sequences (CDS) of the *AmNHX2* and *AmCLC‐a* gene were amplified by PCR using KOD One™ PCR Master Mix‐Blue (TOYOBO, Japan). The DNA fragments of *AmNHX2* and *AmCLC‐a* were then inserted into the plant expression vector p35S‐GFP using the ClonExpress® II One Step Cloning Kit (Vazyme, China), respectively, resulting in the construction of p35S:*AmNHX2‐GFP* and p35S:*AmCLC‐a‐GFP* vectors (Li et al., 2011). The primers used in the experiments are listed in Table S3.

*Agrobacterium* strain GV3101 was used as the carrier to introduce the recombinant plasmid p35S::*AmNHX2*‐*GFP*, p35S:*AmCLC‐a‐GFP*, and the empty plasmid p35S::*GFP* (Tang et al., 2021). A single colony was selected from GV3101 cultures and grown in LB liquid medium containing 35 μg mL^−1^ rifampicin and 50 μg mL^−1^ kanamycin. The cultures were incubated at 28°C with shaking (250 rpm) for 14 h to enhance bacterial growth. The bacterial cells were then collected and washed twice with MS buffer containing 200 μmol L^−1^ acetosyringone to remove excess antibiotics and medium residues. The bacterial suspension was diluted to an optical density (OD600) of 0.6 and left to equilibrate for 1 h. The experiment was conducted on 4–6 leaf‐stage leaves of *Nicotiana benthamiana*. The bacterial suspensions containing the plasmids were injected into the leaves using a syringe. After injection, the leaves were incubated in the dark for 12 h to facilitate T‐DNA integration. The treated plants were then transferred to a growth chamber set to 24°C with a 16‐h light/8‐h dark photoperiod and incubated for an additional 2 days. One plant transformed with the recombinant plasmid and one with the empty plasmid were selected for analysis. The plants were then divided into control and salt treatment groups. The control group was irrigated with tap water, and the salt treatment group was irrigated with 300 mM NaCl, once daily, for 7 consecutive days. Then the contents of Na^+^ and Cl^−^ in the transgenic tobacco leaves were measured using ICP‐MS (7500ce, Agilent) and ion chromatography (ICS‐5000, Thermo), respectively.

The subcellular localization analyses of AmNHX2 and AmCLC‐a also used transgenic tobacco leaves. In brief, *A. tumefaciens* GV3101 transformed with the constructs p35S::*AmNHX2*‐GFP and p35S::*AmCLC‐a*‐GFP were resuspended in a 3% sucrose solution containing 100 μM acetosyringone at an OD600 0.2 and infiltrated in *N. benthamiana* leaves. Subcellular localization was examined 48 h after transformation by confocal laser scanning microscopy (Zeiss LSM 780, Germany).

### Generation of *AmSCAMP1* transgenic *A. thaliana*, TEM observation on vesicles and salt tolerance analyses

The coding sequences (CDS) of the *AmSCAMP1* gene were amplified by PCR using KOD One™ PCR Master Mix‐Blue (TOYOBO, Japan). The DNA fragments of *AmSCAMP1* were then inserted into the plant expression vector p35S‐GFP using the ClonExpress® II One Step Cloning Kit (Vazyme, China), resulting in the construction of p35S:AmSCAMP1‐GFP vectors (Li et al., 2011). The primers used in the experiments are listed in Table S3. The p35S:AmSCAMP1‐GFP was introduced into the *Agrobacterium* strain GV3101, and the *Agrobacterium* strain harboring our vector was transformed into *Arabidopsis thaliana* wild‐type Col‐0 according to a reported protocol (Zhang et al., 2023). Transgenic seedlings were selected on 1/2 MS medium containing 35 mg L^−1^ hygromycin. A T3 homozygous AmSCAMP1‐overexpressing line was obtained for subsequent experiments. TEM observations on vesicles in transgenic *A. thaliana* were performed based on the above‐mentioned methods. Salt tolerance analyses of *AmSCAMP1* transgenic *A. thaliana* include the root length, fresh weight, root Na^+^ flux, and Na^+^ content in the whole plant of the Col‐0 and transgenic *A. thaliana* (OE2 and OE3) under NaCl treatment. Here, the above‐mentioned ICP‐MS method was used to measure Na^+^ content. While Na^+^ fluxes from the root tips were measured using the above‐mentioned NMT method. At least three samples were used for each measurement in flux analysis.

### Real‐time quantitative PCR (RT‐qPCR) analysis of ion transport gene

Relative expression levels of ion transport genes (*AtSOS1*) in *AmSCAMP1* transgenic *A. thaliana* (OE2 and OE3) were assessed by RT‐qPCR. Total RNA was extracted using FastPure Universal Plant Total RNA Isolation Kit (Vazyme, China). DNA‐free RNA was used to synthesize the first strand of cDNA with the HiScript®‐III 1st Strand cDNA Synthesis Kit (Vazyme, China). Primers for RT‐qPCR are shown in Table S3. Then, RT‐qPCR was performed in a CFX384 Touch Real‐Time PCR Detection System (Biorad, USA) with ChamQ Universal SYBR qPCR Master Mix (Vazyme, China), and the procedure followed the reagent and instrument specifications. The relative expression level of *AtSOS1* was calculated by the 2^−∆∆CT^ method (Livak & Schmittgen, 2001) and by *AtActin*, a reference gene in *A. thaliana*, respectively.

### Protein–DNA interaction prediction

Protein–DNA complex structures between AmHAT2 and the predicted HD‐ZIP binding motifs in the promoters of *AmCLC‐a* and *AmSCAMP1* were predicted using the AlphaFold3 online platform (https://alphafoldserver.com) with automatically generated random seeds. The resulting models were visualized using ChimeraX v1.12 (Pettersen et al., 2021).

### Dual‐luciferase reporter assay

For transient Dual‐Luciferase Reporter assays, promoter fragments of *AmCLC‐a* and *AmSCAMP1* were cloned into the pGreenII 0800‐LUC reporter vector. The CDS of *AmHAT2* was inserted into the pGreenII 62‐SK effector vector. Agrobacterium‐mediated transient co‐expression assays were performed in *Nicotiana benthamiana* leaves. Luminescence signals were detected 48 h after infiltration using the IVIS Lumina III imaging system (PerkinElmer, Waltham, MA, USA).

### Statistical analysis

Values in figures were expressed as means ± SE. The statistical significance of the data was analyzed using one‐way ANOVA in SPSS 23.0 and compared using Duncan's multiple range test at a significance level of *P* < 0.05.

## AUTHOR CONTRIBUTIONS

M‐YW and H‐LZ designed the experiments. LS, HL, Z‐JG, and M‐YW performed and analyzed the data. M‐YW wrote the paper. H‐ZH, L‐DZ, D‐NM, Z‐JS, H‐CT, X‐XW, X‐YZ, and H‐LZ gave the suggestions. H‐LZ, M‐YW, L‐HZ, and X‐Y‐H revised this paper. All authors have read and approved the manuscript.

## CONFLICT OF INTEREST

The authors declare that they have no conflict of interest in this work.

## Supporting information


**Figure S1.** Application of laser capture microdissection for isolation of salt gland in *A. marina* leaf sections. Young leaves were fixed, embedded in optimum cutting temperature compound, and 20 μm thick sections were produced. Salt gland cells were easily distinguishable from other cells by their morphological features. (a–d) Pictures show the harvest process of salt gland cells from gland‐rich epidermis including before laser shot (a), selected area (b), after laser shot (c) and harvested cells (d). Scale bars of a, b and c = 40 μm. Scale bar of d = 10 μm.
**Figure S2.** Volcano plot showing differential gene expression levels. Up‐ and down‐regulated genes are represented by red and green symbols. Genes with no significant expression change are represented by blue symbols.
**Figure S3.** Most highly enriched GO terms of DEGs after NaCl treatment. The x‐axis represents the number of DEGs in the enriched groups. The y‐axis represents the different functional groups. The enriched GO terms of up‐regulated DEGs are marked in red, and those of down‐regulated DEGs are marked in green. The number outside the bar represents the number of DEGs that are up‐ or down‐regulated.
**Figure S4.** Subcellular localizations of AmNHX2 and AmCLC‐a in tobacco epidermis cells. Channel from left to right are green fluorescent protein (GFP) fluorescence, bright‐field and merged images of tobacco leaves. Scale bars represent 50 μm.

**Table S1.** Summary of sequence assembly using Illumina sequencing. CK1‐3: 0 mM NaCl; NaCl1‐3: plant treated with 400 mM NaCl.
**Table S2.** Differentially expressed genes (DEGs) in salt glands between the control and 400 mM NaCl‐treated plants.
**Table S3.** Primer sequences used in functional validation of *AmNHX2*, *AmCLC‐a* and *AmSCAMP1* genes.
**Table S4.** Prediction results of transcription factor AmHAT2 binding sites in the promoters of *AmSCAMP1* and *AmCLC‐a*.

## Data Availability

The data that supports the findings of this study are available in the supplementary material of this article.
