# Supplementary material for: A laser capture microdissection‐based transcriptome analysis on salt glands highlights salt secretion mechanism in mangrove plant Avicennia marina
Source: Plant J. 2026 Oct 7;128(1):e71143. doi: 10.1111/tpj.71143 (PMC13646480; doi:10.1111/tpj.71143)
Supplement: Supplementary file 1 [file TPJ-128-0-s001.docx]

**Fig. S1** Application of laser capture microdissection for isolation of salt gland in *A. marina* leaf sections. Young leaves were fixed, embedded in optimum cutting temperature compound, and 20 μm thick sections were produced. Salt gland cells were easily distinguishable from other cells by their morphological features. (a–d) Pictures show the harvest process of salt gland cells from gland-rich epidermis including before laser shot (a), selected area (b), after laser shot (c) and harvested cells (d). Scale bars of a, b and c = 40 μm. Scale bar of d = 10 μm.

**Fig. S2** Volcano plot showing differential gene expression levels. Up- and down-regulated genes are represented by red and green symbols. Genes with no significant expression change are represented by blue symbols.

**Fig. S3** The most highly enriched GO terms of DEGs after NaCl treatment. The x-axis represents the number of DEGs in the enriched groups. The y-axis represents the different functional groups. The enriched GO terms of up-regulated DEGs are marked in red, and those of down-regulated DEGs are marked in green. The number outside the bar represents the number of DEGs that are up- or down-regulated.

**Fig. S4** Subcellular localizations of AmNHX2 and AmCLC-a in tobacco epidermis cells. Channel from left to right are green fluorescent protein (GFP) fluorescence, bright-field and merged images of tobacco leaves. Scale bars represent 50 μm.
