# Supplementary material for: A laser capture microdissection‐based transcriptome analysis on salt glands highlights salt secretion mechanism in mangrove plant Avicennia marina
Source: Plant J. 2026 Oct 7;128(1):e71143. doi: 10.1111/tpj.71143 (PMC13646480; doi:10.1111/tpj.71143)
Supplement: Supplementary file 2 [file TPJ-128-0-s002.zip › tpj71143-sup-0002-TableS1-S4@Supplemental tables.docx]

**Table S1** Summary of sequence assembly using Illumina sequencing. CK1-3: 0 mM NaCl; NaCl1-3: plant treated with 400 mM NaCl.

| Sample | Raw reads | Clean reads | Clean bases | Error (%) | Q20 (%) | Q30 (%) | GC (%) |
| --- | --- | --- | --- | --- | --- | --- | --- |
| CK1 | 47,709,494 | 45,998,650 | 6.9G | 0.04 | 93.98 | 88.95 | 42.29 |
| CK2 | 44,827,578 | 42,760,342 | 6.41G | 0.04 | 91.74 | 85.03 | 40.53 |
| CK3 | 70,927,614 | 65,567,200 | 9.84G | 0.07 | 88.1 | 81.82 | 39.8 |
| NaCl1 | 46,797,424 | 44,783,986 | 6.72G | 0.04 | 92.3 | 85.74 | 41.57 |
| NaCl2 | 44,315,388 | 41,709,546 | 6.26G | 0.05 | 91.28 | 84.59 | 40.28 |
| NaCl3 | 46,385,530 | 43,979,340 | 6.60G | 0.04 | 92.14 | 85.65 | 41.54 |

**Table S2** Differentially expressed genes (DEGs) in salt glands between the control and 400 mM NaCl-treated plants.

**Table S3** Primer sequences used in functional validation of *AmNHX2*, *AmCLC-a* and *AmSCAMP1* genes.

| Primer name | Sequence (5' to 3') |
| --- | --- |
| p35S-AmNHX2-F | GAACACGGGGGACTCTTGACATGAATGAATCTATCACTGCC |
| p35S-AmNHX2-R | CTCACCATGTCGACTCTAGACTGTTTGGCTTCTTCTTCTT |
| p35S-AmSCAMP1-F | GAACACGGGGGACTCTTGACATGGCCGGCCGTTACGATC |
| p35S-AmSCAMP1-R | CTCACCATGTCGACTCTAGAAACTGCTGCCCTGAGGGCTC |
| pYES2-AmNHX2-F | GGAATATTAAGCTTGGTACCATGAATGAATCTATCACTGCC |
| pYES2-AmNHX2-R | CGCCCTTGCTCACCATGGTCTGTTTGGCTTCTTCTTCTT |
| pYES2-AmCLC-a-F | GGAATATTAAGCTTGGTACCATGGAGGACTTTAACAAGTTAGGAG |
| pYES2-AmCLC-a-R | CGCCCTTGCTCACCATGGTATGCGCCTTCATGCTTCC |
| pYES2-AmSCAMP1-F | GGAATATTAAGCTTGGTACCATGGCCGGCCGTTACGATC |
| pYES2-AmSCAMP1-R | CGCCCTTGCTCACCATGGTAACTGCTGCCCTGAGGGCTC |
| P35S-AmCLC-a-F | GAACACGGGGGACTCTTGACATGGAGGACTTTAACAAGTTAGGAG |
| P35S-AmCLC-a-R | CTCACCATGTCGACTCTAGAATGCGCCTTCATGCTTCC |
| AtSOS1q-F | TCCGGTTGCTGTTGTTGCTT |
| AtSOS1q-R | CAACAATCGCCGTCCCATCA |

**Table S4** Prediction results of transcription factor AmHAT2 binding sites in the promoters of *AmSCAMP1* and *AmCLC-a*.

| Pattern name | Family | Sequence name | Start | Stop | p-value | q-value | Matched sequence |
| --- | --- | --- | --- | --- | --- | --- | --- |
| AmHAT2 | HD-ZIP | AmSCAMP1 | 1926 | 1936 | 3.51E-06 | 0.0419 | TCCAATCATTA |
| AmHAT2 | HD-ZIP | AmCLC-a | 209 | 219 | 6.48E-05 | 0.386 | GGCAATCATTG |
